# The FliI ATPase couples ATP hydrolysis to substrate switching in bacterial flagellar type-III secretion

**DOI:** 10.1128/mbio.02354-25

**Published:** 2025-12-05

**Authors:** Rosa Einenkel, Caroline Kühne, Mario Delgadillo‑Guevara, Lasse Hallenga, Christian Goosmann, Marc Erhardt

**Affiliations:** 1 Humboldt-Universität zu Berlin, Institute of Biology 9373, Berlin, Germany; 2 Department of Biochemistry and Pharmacology, Bio21 Molecular Science and Biotechnology Institute, The University of Melbourne 590806, Melbourne, Victoria, Australia; 3 Max Planck Institute for Infection Biology 28260, Berlin, Germany; 4 Max Planck Unit for the Science of Pathogens 528111, Berlin, Germany; University of Utah, Salt Lake City, Utah, USA

**Keywords:** bacterial flagellum, type III protein secretion, ATPase, *Salmonella enterica*

## Abstract

Bacterial flagella are assembled by a specialized type-III secretion system that exports structural subunits in a defined order. While the ATPase FliI is known to couple ATP hydrolysis to substrate translocation, its role in the transition between early and late secretion stages has remained unclear. Here, we systematically analyzed *Salmonella enterica* strains with mutations in the catalytic domain of FliI and found that early substrate export and hook-basal body formation can proceed with minimal FliI ATPase activity, whereas efficient triggering of the substrate specificity switch and late substrate export requires near-wild-type activity levels. Mutant strains showed delayed gene expression from class 3 promoters, prolonged early secretion, and impaired flagellar filament assembly, despite normal FliI localization and oligomerization. These findings support the involvement of FliI in controlling the temporal dynamics of flagellar assembly. We propose that FliI contributes to substrate switching, ensuring robust and orderly function of the flagellar type-III secretion system. This study highlights the multifaceted role of the type-III secretion system ATPase in optimizing the efficiency and robustness of flagellum assembly and establishes a mechanistic link between ATPase activity and substrate switching in type-III secretion systems.

**IMPORTANCE:**

The ordered export of substrates by bacterial type-III secretion systems is essential for the assembly of complex surface structures such as the flagellum, yet the mechanisms that control the timing of substrate switching remain poorly understood. The flagellar type-III secretion system ATPase FliI has previously been implicated in coupling ATP hydrolysis to activation of the proton motive force-driven export apparatus, although its precise role in secretion remains incompletely understood. Here, we show that FliI contributes to the transition from early to late substrate export during flagellar biogenesis. Using mutants of the catalytic domain of FliI in *Salmonella*, we demonstrate that minimal ATPase activity is sufficient to support early export, whereas efficient substrate switching and late-stage flagellar assembly require near-wild-type activity levels. These findings highlight the multifaceted roles of FliI during flagellar assembly beyond its proposed function in activating the flagellar type-III secretion system and demonstrate that the ATPase is critical for coordinating the transition from early to late substrate export, linking ATPase activity to substrate switching in type-III secretion systems.

## INTRODUCTION

**Fig 1:**
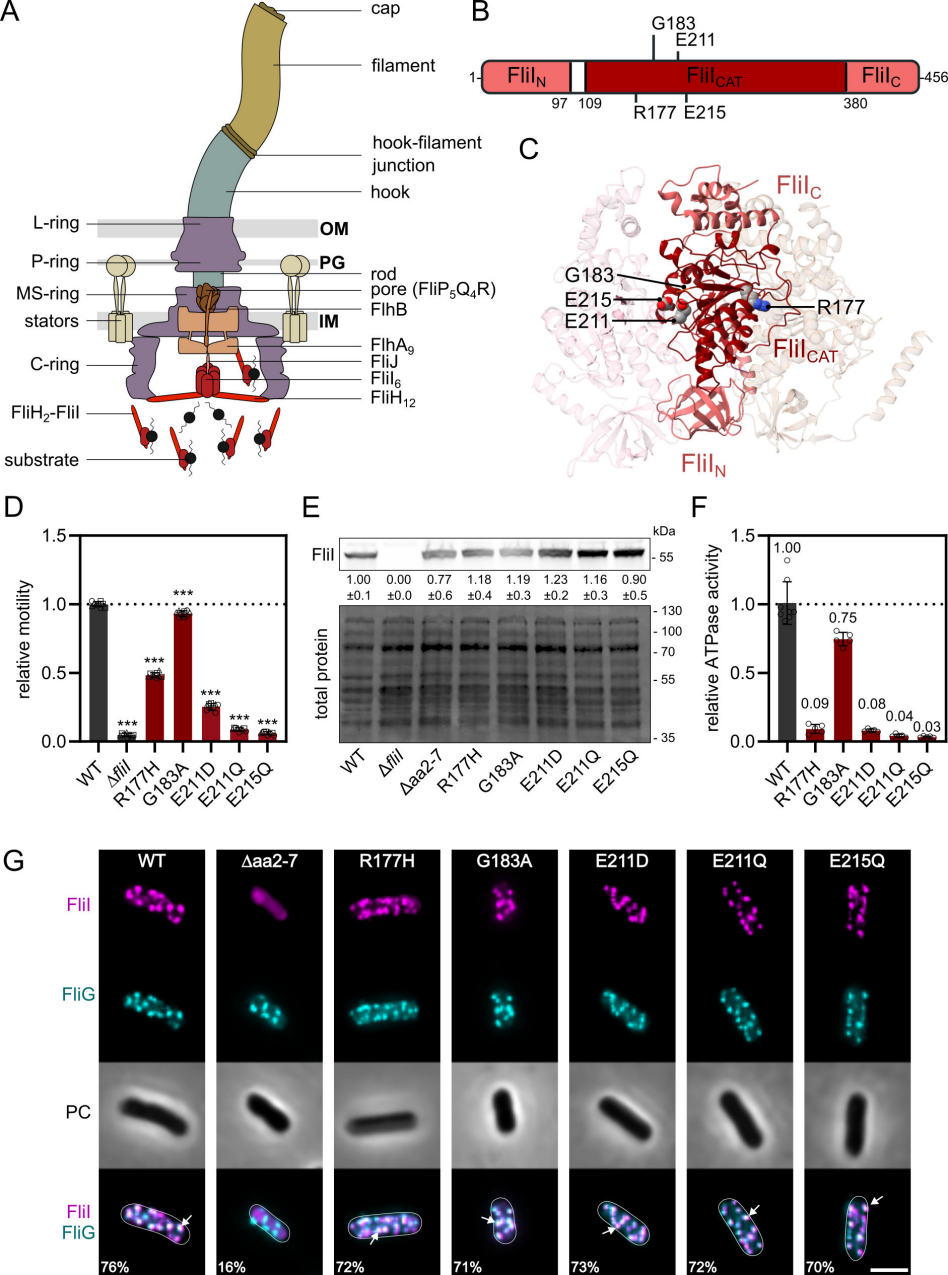
FliI is required for flagellar motility and efficient assembly of the flagellar export apparatus. (**A**) Schematic of a gram-negative bacterial flagellum and the flagellar type-III secretion system (fT3SS). The flagellar motor spans the inner membrane (IM), periplasmic space including the peptidoglycan (PG), and outer membrane (OM), and assembles an external filament via a central secretion channel. The cytoplasmic ATPase complex (FliHIJ) delivers substrates and activates the export gate through ATP hydrolysis. (**B**) Domain organization of FliI and location of investigated residues within FliI_CAT_. (**C**) Atomic model of three subunits of the *Salmonella enterica* FliI hexamer, viewed from the inner channel of the ring. The FliI monomer (PDB ID: 5B0O) was modeled onto the hexameric arrangement of the homologous T3SS ATPase EscN (PDB ID: 6NJO) (17, 18). The positions of residues analyzed in this study (R177, G183, E211, and E215) are highlighted on the central subunit. The three structural domains FliI_N_, FliI_CAT_, and FliI_C_ are shown in distinct shades of red. (**D**) Relative motility of wildtype (WT) and *fliI* mutants analyzed using soft-agar motility plates, quantified after 4–5 h incubation at 37°C. Diameters of the motility halos were measured using Fiji and normalized to the WT. Bar graphs represent the mean of at least three biological replicates with SD error bars. Replicates are shown as individual data points. ***P < 0.001 (ordinary one-way analysis of variance [ANOVA] with Dunnett’s multiple comparison test vs WT). (**E**) Western blot analysis of FLAG-tagged FliI in the cellular fraction. Experiment done in three biological replicates, separated by SDS-PAGE and immunoblotted using anti-FLAG. Numbers indicate the amount of FLAG-tagged FliI normalized to the total protein and to the WT. (**F**) Relative ATPase activity of WT and FliI mutants analyzed using purified proteins and the malachite green assay, quantified after 30 min incubation at 37°C. Release of free phosphate was quantified at 640 nm and normalized to the WT. Bar graphs represent the mean of at least three independent measurements with SD error bars. Individual measurements shown as data points. (**G**) Representative microscopy images of WT and *fliI* mutants. FliI-HaloTag labeled with tetramethylrhodamine (TMR)-ligand (magenta), BBs visualized with mNeonGreen-FliG (cyan); PC (Phase Contrast). Percentage of FliI-FliG foci pairs below the colocalization threshold (2 px) is indicated for each strain. Quantifications are based on analyses of >400 cells per strain (see Table S1). White arrows show examples of colocalized foci. Scale bar: 2 µm.

Bacterial flagella are complex nanomachines that play a crucial role in motility, enabling bacteria to navigate through their environment (1). This motility is essential for many bacterial processes, including colonization and adhesion to host cells (2). The flagellum consists of three major structural components: the basal body (BB), the hook, and the filament (Fig. 1A). The BB anchors the flagellum to the bacterial cell envelope and serves as the motor apparatus, generating the rotational force that drives motility (3, 4). The BB is composed of several ring-like structures, including the peptidoglycan-spanning P-ring, the outer membrane L-ring, and the inner membrane MS-ring, which connects to the rotor (3, 4). The rotor, or C-ring, links flagella-mediated motility to the process of chemotaxis and acts as the rotary switch complex (5–7). The stator units generate the rotational force that drives the rotation of the C-ring and hence the flagellar filament (8). The rod extends from the inner membrane MS ring through the periplasm, where it is assembled by the rod cap FlgJ that also has muramidase activity (9). The rod functions as a drive shaft and contains the central channel for protein secretion (10). The hook acts as a flexible joint between the BB and the filament (11), with its length precisely regulated by the molecular ruler FliK to ensure proper motility (12, 13). During assembly, the hook is capped by FlgD, which promotes polymerization of the hook protein (14). The filament, built from tens of thousands of flagellin subunits (FliC or FljB in *Salmonella enterica*), is connected to the hook via the hook-filament junction (FlgKL) and is capped by a pentamer of FliD, a specialized structure that facilitates the assembly of flagellin subunits into a functional filament (15, 16).

Flagellar gene expression follows a hierarchical system that couples assembly status to transcription (19). The master regulator FlhD_4_C_2_ activates transcription from class 2 promoters, which drive expression of genes encoding for hook-basal body (HBB) components as well as regulatory proteins like the flagellar-specific transcription factor σ^28^ and its anti-σ factor FlgM. FlgM coordinates the temporal progression of flagellar gene expression by sequestering and inhibiting σ^28^ until hook completion, when the export apparatus switches from early substrates (rod and hook components) to late substrates (filament components). This transition ensures proper hook length, which is essential for optimal motility (12, 13). At this point, FlgM is recognized as a late substrate and secreted via the flagellar type-III secretion system (fT3SS), freeing σ^28^ to finally initiate transcription from class 3 promoters (20–22). Class 3 promoters drive expression of genes encoding late structural and functional proteins, including the flagellins, the stator units, and key components of the chemotaxis system (23).

The secretion of axial proteins is tightly regulated and mediated by the fT3SS, which is responsible for the translocation of most extra-cytoplasmic flagellar components from the bacterial cytoplasm to the site of assembly, a process that is mainly powered by the proton motive force (24). The fT3SS, as part of the BB, consists of the membrane-embedded export gate (FlhA and FlhB) and secretion pore (FliP, FliQ, and FliR), as well as a cytoplasmic ATPase complex (FliH, FliI, and FliJ) (25). FlhA is a central component of the export gate, consisting of an N-terminal, transmembrane domain, and a C-terminal, cytoplasmic domain (FlhA_C_). FlhA_C_ assembles into a homo-nonameric ring that functions as a docking platform for flagellar substrates, thereby organizing their ordered export during assembly (26). Studies have shown that the FlhA_C_ ring can adopt at least two conformational states: a closed state that favors early substrates and an open state that promotes late-substrate export. The transition between these states is a prerequisite for the substrate specificity switch, a key feature of the fT3SS. This remodeling of FlhA_C_ is mediated by interactions of its flexible linker (FlhA_L_) with the neighboring subunit and is triggered by interactions of FliK with the C-terminal domain of FlhB once the hook reaches its optimal length of ~55 nm (27–30). A recent study suggested that the cytoplasmic ATPase complex, specifically FliH and FliI, supports the efficient remodeling of FlhA_C_ (31).

FliI, a Walker-type AAA^+^-ATPase with two highly conserved motifs for nucleotide binding (Walker A) and ATP hydrolysis (Walker B), forms a hexameric ring structure at the base of the flagellum with FliJ as the central stalk, anchored to the BB by 12 FliH molecules through interactions with the motor protein FliN (17, 32–36). The ATPase complex is thought to couple ATP hydrolysis to the activation of the export gate through interactions of FliJ with FlhA_L_ to ensure efficient and robust protein export (37). Notably, under specific *in vitro* conditions, ATP hydrolysis alone has been shown to drive substrate export in the absence of bulk proton motive force, underscoring the energetic contribution of the fT3SS ATPase complex (38). While a previous study had proposed a potential interaction between the second messenger c-di-GMP and the fT3SS ATPase FliI (39), the exact role of this interaction remains unclear. Furthermore, heterotrimers of 2 FliH and 1 FliI are thought to play an important role in the dynamic delivery of substrates to the export gate, ensuring the timely assembly of the flagellar components (40). Based on the known functions of the evolutionarily related ATPase InvC from the virulence-associated T3SS, as well as insights from mathematical modeling, FliI has been proposed to play roles in substrate unfolding and in dissociating substrates from their cognate chaperones (22, 41). However, although extensively studied, the exact molecular mechanisms of the fT3SS ATPase complex remain incompletely understood (42).

In this study, we dissected the role of the flagellar ATPase FliI by generating targeted point mutations in its ATPase domain. Through a combination of mutagenesis and functional assays, we identified mutants that exhibited selective defects in the secretion of late substrates and in the substrate specificity switch, while early substrate export and hook assembly remained largely unaffected. These results demonstrate that only minimal ATPase activity is required for early stages of flagellar assembly, whereas efficient triggering of the transition to late-stage export depends on near-wild-type (WT) activity levels. Our findings support a model in which ATP hydrolysis by FliI not only powers secretion but also actively supports the substrate switching mechanism by stabilizing the transition of FlhA_C_ from the closed to the open conformation, thereby ensuring the temporal fidelity and efficiency of flagellar biogenesis.

## RESULTS

### FliI catalytic domain mutants impair motility but retain localization and hexamer formation

The Walker-type AAA+ ATPase FliI from *S. enterica* plays a crucial role in flagellar assembly by ensuring efficient export of substrates through the fT3SS. FliI consists of three domains: the N-terminal domain (FliI_N_), required for FliI ring formation and interactions with FliH; the catalytic domain (FliI_CAT_), containing both Walker A and Walker B motifs for ATP binding and hydrolysis; and the C-terminal domain (FliI_C_), which undergoes conformational changes upon ATP hydrolysis and interacts with FliJ (Fig. 1B) (35, 42).

To investigate the molecular function of FliI, we generated point mutations within FliI_CAT_ that had been implicated in the catalytic activity of FliI of *S. enterica* or suggested to play regulatory roles in FliI of *Pseudomonas fluorescens* (*P. fluorescens*) (35, 39, 43). All mutations were introduced into the native *S. enterica* genomic location of *fliI* to maintain native expression conditions of the ATPase. Three substitutions (R177H, G183A, and E215Q) were selected because homologous residues in *P. fluorescens* FliI had been reported to affect ATPase activity and cyclic di-GMP binding (39). In *S. enterica* FliI, these residues occupy functionally important positions: R177 and E215 are located at the monomer-monomer interface, with structural studies also implicating E215 in catalysis, while G183 is the first conserved glycine in the Walker A motif. To our knowledge, these residues have not been functionally characterized in *S. enterica* before. Although G183 is not directly contacting ADP (35), substitution of the homologous residue in *P. fluorescens* modestly reduced ATPase activity, making G183A a useful variant to probe mild perturbations in nucleotide binding. In addition, we included two previously characterized mutants of the catalytic glutamate in the Walker B motif (E211D and E211Q), known to drastically reduce ATPase activity—by about 100-fold and almost completely, respectively—resulting in impaired motility and substrate secretion (43).

We assessed the effect of *fliI* point mutations on swimming motility using a soft-agar motility assay (Fig. 1D). As anticipated, the *fliI* deletion mutant (Δ*fliI*) was non-motile, consistent with previous reports (24). The R177H mutant displayed 48% motility compared to the WT, while the E215Q mutant was non-motile. The Walker A motif mutant G183A exhibited a slight reduction in motility to 93% of the WT levels. The catalytic glutamate mutants E211D and E211Q showed 25% motility or loss of motility, respectively. These phenotypes suggest defects in flagellar assembly, prompting further investigation into how these specific mutations impair motility. To assess whether the observed phenotypes could be attributed to altered protein abundance, we examined cytoplasmic levels of chromosomally expressed FLAG-tagged FliI variants by immunoblotting. FliI protein levels were comparable across the WT and all mutant strains (Fig. 1E), suggesting that the observed phenotypes are not due to differences in protein expression or stability *in vivo*.

ATP hydrolysis by the cytoplasmic ATPase FliI is thought to activate the flagellar export apparatus (42). Specifically, ATP hydrolysis induces conformational changes in FliI_C_, which in turn drive rotation of FliJ. Subsequent interactions between FliJ and FlhA_L_ induce conformational rearrangements and opening of the polypeptide and proton channels of the export gate. Mutations that impair ATP binding or catalysis are therefore expected to compromise motility by reducing the efficiency of protein secretion. To test how the selected substitutions affect enzymatic activity, we purified each FliI variant and measured ATP hydrolysis using a malachite green assay.

We measured the ATPase activities of each protein after 30 min of incubation with ATP and normalized the values to the WT protein (Fig. 1F). Strikingly, the R177H substitution strongly impaired activity to 9% of WT, despite the mutant strain retaining 50% motility. We note that purification of the R177H variant yielded lower amounts of soluble protein, suggesting that this variant is more unstable *in vitro*. As this variant is prone to aggregation *in vitro*, we therefore cannot exclude that the R177H variant retains higher ATPase activity *in vivo*. Importantly, however, western blot analysis of chromosomally expressed FliI confirmed that *in vivo* protein levels of the R177H variant were comparable to WT (Fig. 1E), indicating that its phenotype indeed cannot be fully explained by reduced expression or stability in the cell. Consistent with previous observations in *P. fluorescens*, the nucleotide-binding mutant G183A retained ~75% of WT activity, suggesting that ATP binding is only mildly hindered in this mutant (39). The mutants of the catalytic glutamate, E211D and E211Q, displayed similar activities as previously described (43). Specifically, their activities were reduced to ~8% (E211D) and ~4% (E211Q) of the WT, consistent with their reported roles in catalysis. The E215Q substitution almost completely abolished ATP hydrolysis (3% of WT), consistent with its implication in ATP hydrolysis (35). Together, these data demonstrate that the tested substitutions in the catalytic domain have distinct effects on ATP hydrolysis, ranging from mild reduction to near-complete loss of activity. Notably, the degree of ATPase reduction does not directly correlate with motility phenotypes: mutants with very low activity (R177H, E211D, E211Q, and E215Q) exhibited markedly different levels of motility.

To investigate whether the phenotypes observed in the FliI mutants were caused by subcellular mislocalization of the ATPase, we performed *in vivo* fluorescence microscopy. We constructed strains that express FliI with a C-terminal HaloTag fusion and co-express the C-ring component FliG with an N-terminal mNeonGreen fusion to visualize colocalization with the BB. To synchronize flagellar biosynthesis, we replaced the native promoter of the *flhDC* master regulator operon with an anhydrotetracycline (AnTc) inducible P*_tetA_* promoter (44). We labeled FliI-HaloTag with the cell-permeant ligand tetramethylrhodamine (TMR) and performed fluorescence microscopy on live cells (Fig. 1G). We then quantified the spatial colocalization between foci detected in the respective fluorescence channels by calculating pairwise distances between detected spots across individual cells. We defined fluorescent spots as colocalized when their distance was below a predefined threshold (2 px or ~144 nm) and determined the fraction of colocalized foci pairs by dividing the number of colocalized pairs by the total number of detected pairs within a maximal distance (30 px or ~2 µm).

In WT cells, FliI strongly colocalized with FliG at the BB, forming distinct overlapping foci with 76% of foci pairs below the colocalization threshold (Fig. 1G). As a negative control, we analyzed the FliI ∆aa2-7 mutant, which has been previously shown to be deficient in hexamer formation (45) and interaction with FliH (46). This variant displayed a diffuse cytoplasmic localization, with no distinct FliI foci visible by microscopy. Because our analysis pipeline detects local intensity maxima, it still identified foci pairs, but only 16% met the colocalization criteria. Consistent with this, the average number of detected foci pairs per cell dropped from 2.4 in the WT to 0.7 in the Δaa2-7 mutant, and the distribution of foci distances was substantially broader compared to all other strains (Table S1 and Fig. S1A). These results confirm that FliI ∆aa2-7 fails to localize to the BB, likely due to its inability to properly form hexamers and disrupted interaction with FliH. Importantly, the FliI point mutants (R177H, G183A, E211D, E211Q, and E215Q) localized to the BB with a frequency similar to the WT, as indicated by the presence of discrete FliI foci (Fig. 1G). The percentage of colocalized foci pairs was 72% for R177H, 71% for G183A, 73% for E211D, 72% for E211Q, and 70% for E215Q. These results suggest that the FliI point mutations do not substantially impair the subcellular targeting of FliI. Additionally, we confirmed that proper localization of FliI depends on FliH and FliN but not on FliJ or FlhA, as previously reported (Fig. S1B) (36).

Efficient ATP hydrolysis by FliI requires oligomerization into a hexameric ring, as ATPase activity displays positive cooperativity with protein concentration (33). Consistently, mutants such as ∆aa2-7 that are defective in hexamerization exhibit reduced ATPase activity (45). To assess whether the FliI point mutants affect hexamer formation, we performed *in vivo* site-specific photo-crosslinking of FliI variants containing a photoactivatable pBpa residue (FliI*) at the FliI-FliI interface (Fig. S1C). In this assay, we expressed FLAG-tagged FliI* variants constitutively from a plasmid in a ∆*fliI* background, which can result in protein levels that differ from the chromosomal context. We observed UV-dependent formation of higher-molecular-weight FliI adducts for WT FliI* and all its point mutants studied here, indicating that these variants retain the ability to oligomerize (Fig. S1D). In contrast, FliI* ∆aa2-7 exhibited only a faint high molecular weight band, suggesting premature oligomerization in the cytoplasm, likely due to the high-level plasmid expression of FliI*. Importantly, the reduced level of cross-links compared to the WT confirms that hexamer formation in this mutant is defective but may still occur at low levels under the experimental conditions. Taken together, these results demonstrate that, while the mutations in FliI impair motility to varying degrees, they do not substantially alter its subcellular localization or ability to form oligomers. Thus, the observed motility defects in the FliI point mutants may instead reflect disruptions in the secretion activity of the fT3SS.

### FliI facilitates secretion of late flagellar substrates

The fT3SS mediates the assembly of the axial structures of the flagellum through two distinct secretion modes: early and late. Upon hook completion, the fT3SS undergoes conformational changes, allowing recognition and export of late substrates (13, 29). To determine whether the motility defects of the FliI point mutants result from impaired secretion, we systematically analyzed early and late substrate export in these strains.

First, we confirmed FliI localization in strains deleted for components that lock the fT3SS in either the early (∆*flgBC* [proximal rod], ∆*flgE* [hook]) or late (∆*flgKL* [hook-filament junction]) stages of flagellar assembly (Fig. S1B). Using fluorescence microscopy, we revealed that FliI colocalized with the BB in both secretion states, indicating its presence at the BB throughout the flagellar assembly process.

**Fig 2:**
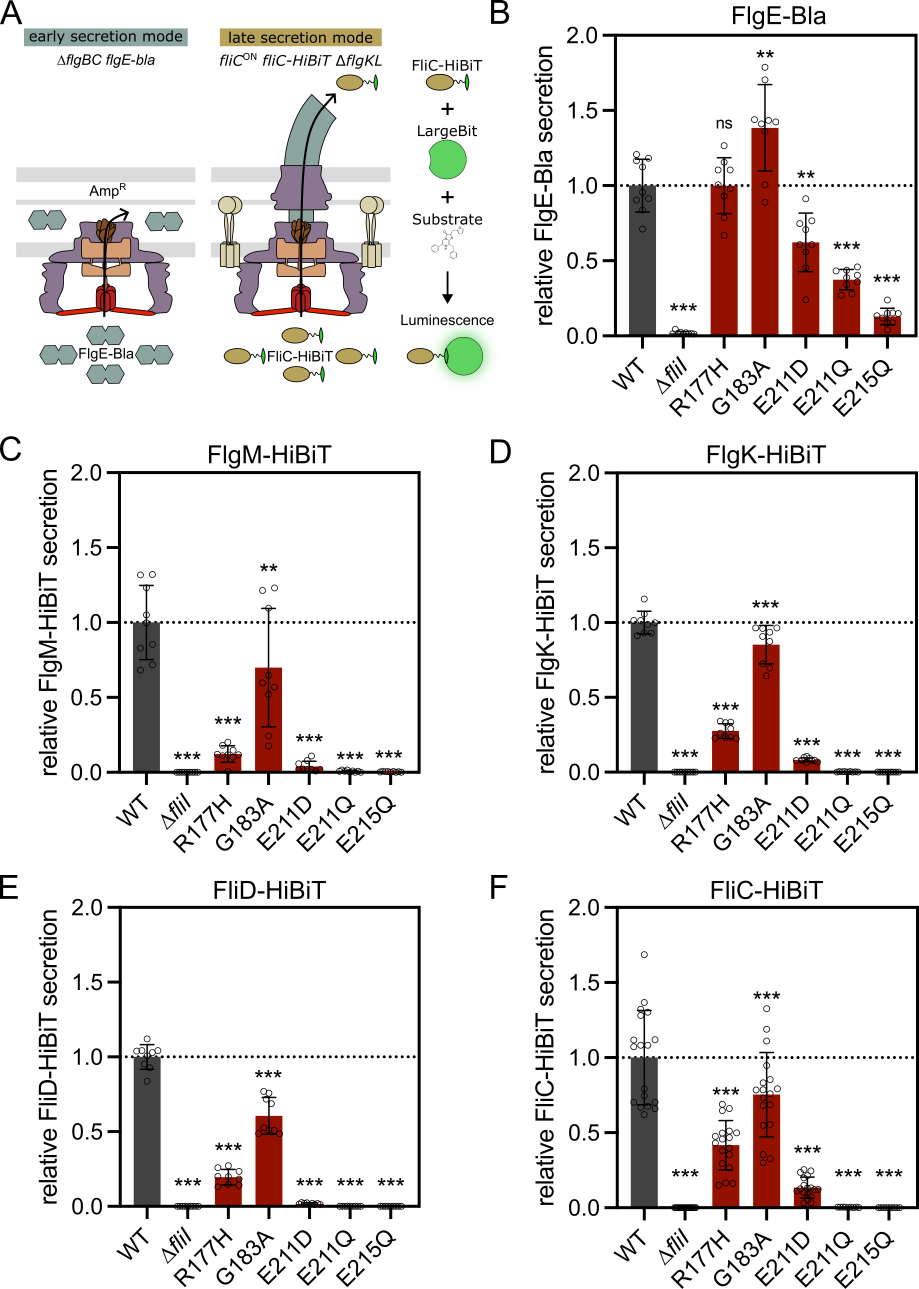
FliI facilitates secretion of late substrates. (**A**) Schematic of experimental set-ups to quantify secretion of early (FlgE-Bla) and late substrates (example for FliC-HiBiT). Left: early substrate secretion was measured in strains lacking proximal rod components, allowing secretion of FlgE-Bla into the periplasm, where it confers ampicillin-resistance (Amp^R^), proportional to the amount of secreted FlgE-Bla. Right: late substrate secretion was quantified using the split Luciferase NanoBit. Strains were locked in FliC production (*fliC*^ON^) and lacked the hook-filament junction to prevent filament assembly (∆*flgKL*). Cells expressed a translational fusion of FliC to HiBiT. The addition of LargeBit and substrate to the culture supernatant enabled luminescence detection proportional to secreted FliC-HiBiT levels. (**B**) Quantification of early substrate (FlgE-Bla) secretion. Relative ampicillin resistance normalized to WT. Bars represent means ± SD from at least three independent experiments; **P < 0.01 and ***P < 0.001; ns, not significant (ordinary one-way ANOVA with Dunnett’s multiple comparison test vs WT). (**C–F**) Quantification of late substrate secretion, normalized to the OD_600_ and to the WT. Bars represent means ± SD from at least three independent experiments; ***P < 0.001 (ordinary one-way ANOVA with Dunnett’s multiple comparison test vs WT).

To analyze the secretion efficiency of early substrates in FliI point mutants, we employed a hook protein/β-lactamase (FlgE-Bla) reporter system (Fig. 2A) (47, 48). The deletion of the rod components FlgBC leads to the secretion of flagellar substrates into the periplasm (49), resulting in the ability to resist ampicillin in correlation to the quantity of secreted FlgE-Bla. Therefore, we analyzed the ability of the FliI mutants to grow in the presence of increasing ampicillin concentrations and determined the concentration of ampicillin leading to inhibition of bacterial growth to half of its maximum (IC_50_ [Amp], Fig. S2A) and then normalized it to WT levels (Fig. 2B). We used a mutant lacking the MS-ring and therefore the entire fT3SS (∆*fliF*) as a negative control, representing the basal level of resistance against ampicillin (Fig. S2A).

Deletion of *fliI* resulted in a severe reduction of early substrate secretion to 2% of WT levels, similar to the ∆*fliF* control strain (1%), consistent with previous observations (24). Among the point mutants, G183A exhibited the highest secretion efficiency, with an IC_50_ of 356 µg/mL corresponding to 138% of WT levels, indicating that a mild reduction of ATPase activity does not affect the secretion of early substrates. The R177H mutant displayed secretion comparable to WT (IC_50_ of 257 µg/mL compared to 256 µg/mL for the WT), despite significantly reduced motility and ATPase activity. Thus, both R177H and G183A were proficient in early substrate secretion, with G183A even showing modestly increased levels, which we interpret as a subtle shift in the balance toward extended early export. In contrast, substitutions at catalytic residues reduced secretion efficiency to varying degrees. E215Q exhibited the most severe defect, with only 13% of WT levels, though still 11-fold higher than the ∆*fliF* control. The Walker B motif mutants E211D and E211Q retained partial secretion activity (62% and 37% of WT, respectively), consistent with E211D retaining higher ATPase activity than E211Q. Notably, even the low-activity mutants (E211D, E211Q, and E215Q) retained substantially higher secretion than the ∆*fliF* strain (52-, 31-, and 11-fold, respectively), underscoring that small differences in residual ATPase activity are sufficient to influence the efficiency of early substrate export (Fig. S2A).

For quantification of late substrate secretion, we next employed the Nano-Glo extracellular detection system (Promega) and the split luciferase NanoBit (Fig. 2A) (50, 51). We engineered strains to express late flagellar substrates translationally fused to the small luciferase subunit HiBiT (52). For each substrate, we tested several HiBiT insertion sites and selected the constructs with the best secretion performances. Secreted HiBiT-tagged proteins accumulated in the supernatant (SN) and were detected via luminescence (Fig. 2C through F).

We first examined the secretion of FlgM, one of the earliest late substrates to be exported once the hook reaches its mature length. All mutants with impaired early secretion (∆*fliI*, E211D, E211Q, and E215Q) displayed similarly severe defects in FlgM export, ranging from 0.1% to 7% of WT levels (Fig. 2C). R177H also showed a pronounced reduction (12%), and G183A displayed a moderate decrease to ~70% secreted FlgM levels compared to the WT. These results suggest that the reduced ATPase activity in these mutants becomes more apparent at the secretion stage of late substrate, compared to early export.

The subsequent substrates, FlgK and FliD, which form the hook-filament junction and filament cap, followed the same overall trend observed for FlgM. Secretion of FlgK was nearly abolished in Δ*fliI*, E215Q, and E211Q (<0.3% of WT), while E211D, R177H, and G183A retained 8%, 28%, and 85% of WT levels, respectively (Fig. 2D). A similar pattern was observed for FliD-HiBiT (Fig. 2E): export was abolished in Δ*fliI*, E215Q, and E211Q, very low in E211D (2%), and strongly to moderately reduced in R177H (20%) and G183A (61%). These results indicate that secretion defects are general for late flagellar substrates and not restricted to a specific substrate protein.

Whereas the ATPase FliI has been shown to directly interact with substrate-chaperone complexes of the hook-filament junction and filament cap proteins, such an interaction was not observed for FliC and its cognate chaperone FliS (53–55). To test whether the impaired export of FlgK and FliD could reflect defects in these specific interactions rather than a broader deficiency of the fT3SS, we next examined secretion of the flagellin FliC. However, FliC secretion followed the same trend (Fig. 2F). E211D secreted ~14% of WT levels, while Δ*fliI*, E215Q, and E211Q were essentially deficient (≤0.3%). In contrast, G183A and R177H retained 75% and 42% of WT activity, respectively, consistent with their intermediate motility phenotypes.

Western blot analysis of cellular and secreted fractions (Fig. S2B through E) confirmed the luminescence data. Additionally, reduced cellular levels of FliC-HiBiT in most mutants suggested a delayed substrate specificity switch, likely contributing to the impaired secretion phenotypes (Fig. S2E). Hence, secretion of all tested late substrates was consistently reduced across the *fliI* mutants, suggesting a general requirement for FliI in late substrate export. Together, these results are consistent with previous reports that early substrates can be secreted with minimal ATP hydrolysis (43). The increased early substrate secretion in the G183A mutant and the unaffected secretion in the R177H mutant indicate that FliI activity is likely more critical for late substrate export than for early stages of assembly. This is further supported by the observation that all catalysis mutants (E211D, E211Q, and E215Q) retained partial early secretion, despite strongly impaired ATPase activity. Together, these findings suggest that FliI may promote late substrate export secretion either by enhancing transport efficiency or by contributing to the substrate specificity switch.

### ATP hydrolysis by FliI promotes efficient substrate specificity switch

As mentioned above, the observed decrease of late substrate secretion in the *fliI* mutant strains could result either from a defect in the secretion efficiency of late substrates or from a defect or delay in the substrate specificity switch. Before hook completion, the late substrate and anti-σ^28^ factor FlgM inhibits the transcription of genes from class 3 promoters by complexing the flagellar specific σ^28^ (56). The fT3SS undergoes a substrate specificity switch upon complete formation of the HBB, which results in the secretion of FlgM, thereby allowing formation of the σ^28^-RNA-Polymerase holoenzyme and transcription from class 3 promoters (57). Inefficient late substrate secretion would therefore not only result in lower FliC secretion levels but also simultaneously result in a lower abundance of late substrates due to prolonged sequestration of σ^28^ by FlgM.

**Fig 3:**
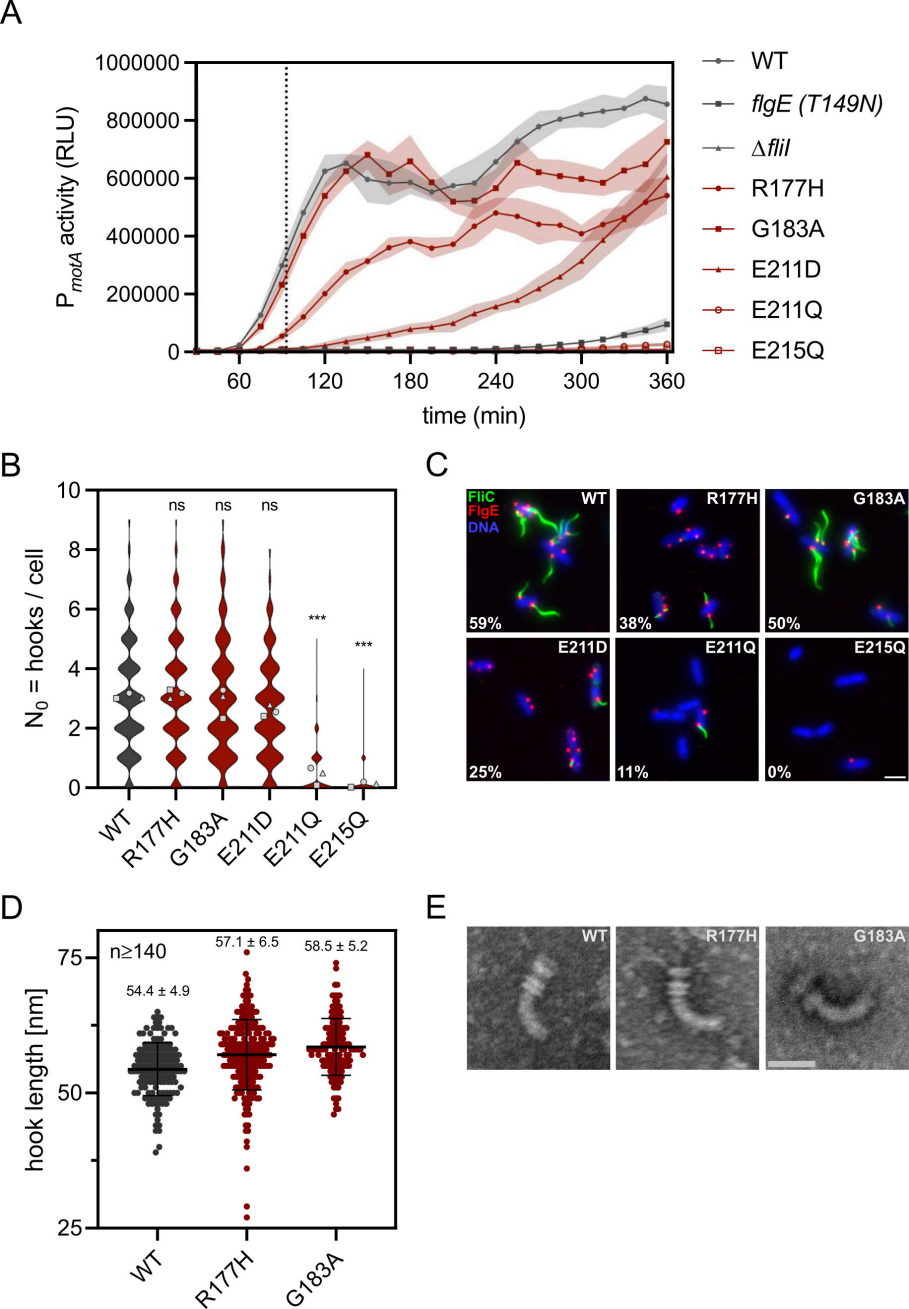
FliI contributes to the substrate specificity switch. (**A**) Time course of P*_motA_* activity (class III gene expression). Luciferase reporter activity was monitored in strains with synchronized flagella synthesis. Shown are the mean relative light units (RLU; lines and symbols) ±SD (transparent lines) measured in six biological replicates. A dashed line indicates half-maximal induction time for *P_motA_* in the WT. (**B**) Quantification of hook number per cell determined by fluorescence microscopy. Violin plots represent the distribution of the data from three biological replicates; symbols represent the mean of the biological replicates. ***P < 0.001; ns, not significant (ordinary one-way ANOVA on the means with Dunnett’s multiple comparison test vs WT). (**C**) Representative fluorescence microscopy images of WT and *fliI* point mutants. Filaments (FliC T237C) labeled with star green maleimide (green), hooks (FlgE_3×HA_) immunostained with α-HA-Alexa555 (red), and DNA counterstained with DAPI. Percentage of hooks with an attached filament indicated in white. Scale bar: 2 µm. (**D**) Hook length measurements from purified HBBs. Symbols represent individual measurements, horizontal black line indicates mean ± SD error bars. (**E**) Representative negative-stain Transmission Electron Microscopy images of purified HBBs. Scale bar = 50 nm.

To address possible delays in the switch mechanism, we monitored gene expression from class 3 promoters using strains with an inducible *flhDC* master operon for synchronized flagella formation and a plasmid harboring the reporter genes *luxCDABE* under control of the class 3 promoter P*_motA_* (58). We found a half-maximal induction time of 93 ± 3 min of the P*_motA_* promoter after *flhDC* induction for the WT (Fig. 3A, dotted line). Furthermore, we determined the relative class 3 gene expression at T_90_ and T_360_ (Fig. S2A). We utilized the previously described slow hook polymerization mutant *flgE*_T149N_ as a control (59, 60). Since the hook polymerization in this mutant takes much longer, the substrate specificity switch, and therefore activation of transcription from class 3 promoters, is delayed.

The G183A mutant displayed the mildest defect in the capability to perform the substrate specificity switch with a relative class 3 gene expression of ~80%. In R177H, P*_motA_* activity was strongly reduced at early timepoints (18% of the WT at T_90_) but recovered to 63% at T_360_, consistent with its reduced FlgM secretion and a delayed, inefficient switch. The catalytic glutamate mutant E211D also showed a gradual increase in promoter activity (3% at T_90_ to 71% at T_360_), reflecting its residual ATPase activity. This delayed yet eventual recovery of P*_motA_* activity in R177H and E211D may be explained by the irreversible nature of the substrate specificity switch: once the export gate transitions to the late conformation and FlgM is secreted, the switch is unable to revert (61). Thus, these mutants ultimately “catch up” to WT promoter activity, even though the switch occurs less efficiently and later in the assembly process. In contrast, the ∆*fliI*, E211Q, and E215Q mutants remained at 1%–3% throughout the experiment, indicating that near-complete loss of ATPase hydrolysis essentially prevents the switch to occur.

However, another possible reason for the observed reduction of class 3 gene expression in the mutant strains could be a reduced number of completed HBBs that assemble per cell. A slower hook polymerization, similar to the *flgE*_T149N_ control strain, and hence a reduced number of completed hooks, would delay the substrate specificity switch. Only upon completion of the HBB does the substrate specificity switch occur, and the anti-σ^28^ factor FlgM will be recognized as a substrate of the fT3SS. Accordingly, a delayed and/or decreased HBB formation frequency, resulting in FlgM remaining inside the cell, would also explain the observed delay in class 3 gene expression.

To address this possible reason for the observed delayed substrate specificity switch in the *fliI* mutants, we determined the number of hooks per cell (Fig. 3B). This was especially interesting for the R177H and G183A mutant, since both mutants did not display a reduction of early substrate secretion but showed reduced class 3 gene expression levels (Fig. 2C and 3A). Furthermore, we determined the number of HBBs with an attached filament (Fig. 3C; Fig. S4). We hypothesized that, if the substrate specificity switch was intact, the ratio of attached filaments to the HBBs would be similar to the WT. On the other hand, an increased number of HBBs without attached filaments would suggest an impairment in the substrate specificity switch. To determine the number of hooks and attached filaments per cell, we simultaneously visualized the hook and filament using strains expressing *flgE*_3×HA_ for immunostaining of the hook and *fliC*_T237C_ for maleimide staining of the filament (Fig. 3C).

Consistent with their early substrate secretion phenotype, R177H and G183A assembled WT-like numbers of HBBs, indicating that HBB formation is intact in these mutants, and their reduced class 3 expression does not result from impaired HBB assembly. The ATPase activity mutant E211D displayed a slight but non-significant reduction of hooks per cell, whereas mutants with near-complete loss of ATPase activity showed clear defects, with over half (E211Q) or the vast majority (E215Q) of cells failing to assemble any HBBs. These findings suggest that even modest differences in residual ATPase activity among the low-activity mutants can have strong effects on HBB formation.

We next assessed filament attachment by simultaneously labeling hooks and filaments (Fig. 3C; Fig. S4). Because filament assembly requires completion of the HBB and a functional substrate specificity switch, we only included cells with hooks in the analysis. The frequency of filament formation followed ATPase activity: WT cells attached filaments to 59% of hooks, while G183A showed a modest reduction to 50%. In contrast, in the R177H mutant, despite normal HBB numbers, we observed attached filaments to only 38% of hooks, indicating an inefficient switch to late secretion mode. The catalytic glutamate mutants E211D and E211Q displayed more pronounced filament assembly deficiencies (25% and 11%, respectively), with E215Q completely failing to assemble filaments despite occasional HBB formation. Thus, the progressive reduction in the fraction of hooks assembling filaments among the mutants supports a role of FliI ATPase activity in enabling the substrate specificity switch.

To further confirm the involvement of the FliI ATPase in the switching mechanism, we measured the hook lengths of the WT and R177H and G183A mutants (Fig. 3D through E). We hypothesized that the delayed substrate specificity switch in the mutants would prolong the operating time of the T3SS in its early substrate secretion mode, resulting in elongated hooks. Therefore, we purified HBB complexes from the WT and the R177H and G183A mutants and measured the lengths of the hooks. Importantly, the lengths of the hooks of both mutants were slightly increased. The WT displayed hooks with an average length of 54.4 ± 4.9 nm, whereas the hooks of the R177H mutant were on average 57.1 ± 6.5 nm. In line with the increased secretion of early substrates (Fig. 2C) in the G183A mutant, the hooks from this mutant were even longer, on average 58.5 ± 5.2 nm. The elongated hooks assembled by the FliI mutants further support an involvement of the fT3SS ATPase FliI in the substrate specificity switch.

Collectively, our results demonstrate that FliI couples ATPase activity to substrate specificity switching of the fT3SS. The introduced point mutations impaired the transition to late substrate secretion, as reflected in reduced class 3 gene expression, fewer hooks with attached filaments, and elongated hooks. These effects are not due to defects in FliI oligomerization or BB localization, as confirmed by *in vivo* photo-crosslinking and fluorescence microscopy. Thus, while low FliI ATPase activity is sufficient to export early substrates, near-WT activity is important for efficient progression to the late assembly mode.

## DISCUSSION

ATP hydrolysis by the FliI ATPase is essential for efficient operation of the fT3SS, but its precise contribution to the temporal regulation of flagellar assembly has remained unclear. In this study, we show that low activity levels of the FliI ATPase are sufficient for early substrate secretion, whereas near-WT activity is required for the substrate specificity switch and efficient export of late substrates. The observed defect in the secretion of late substrates was not limited to FliC, as secretion of other late substrates (FlgM, FlgK, and FliD) was similarly impaired (Fig. S2C), supporting a general role of FliI in facilitating late substrate export. Our analysis of FliI point mutants demonstrates that they exhibit impaired late-stage assembly, with active early substrate export and HBB formation, revealing a role for FliI in supporting the transition to late secretion.

**Fig 4:**
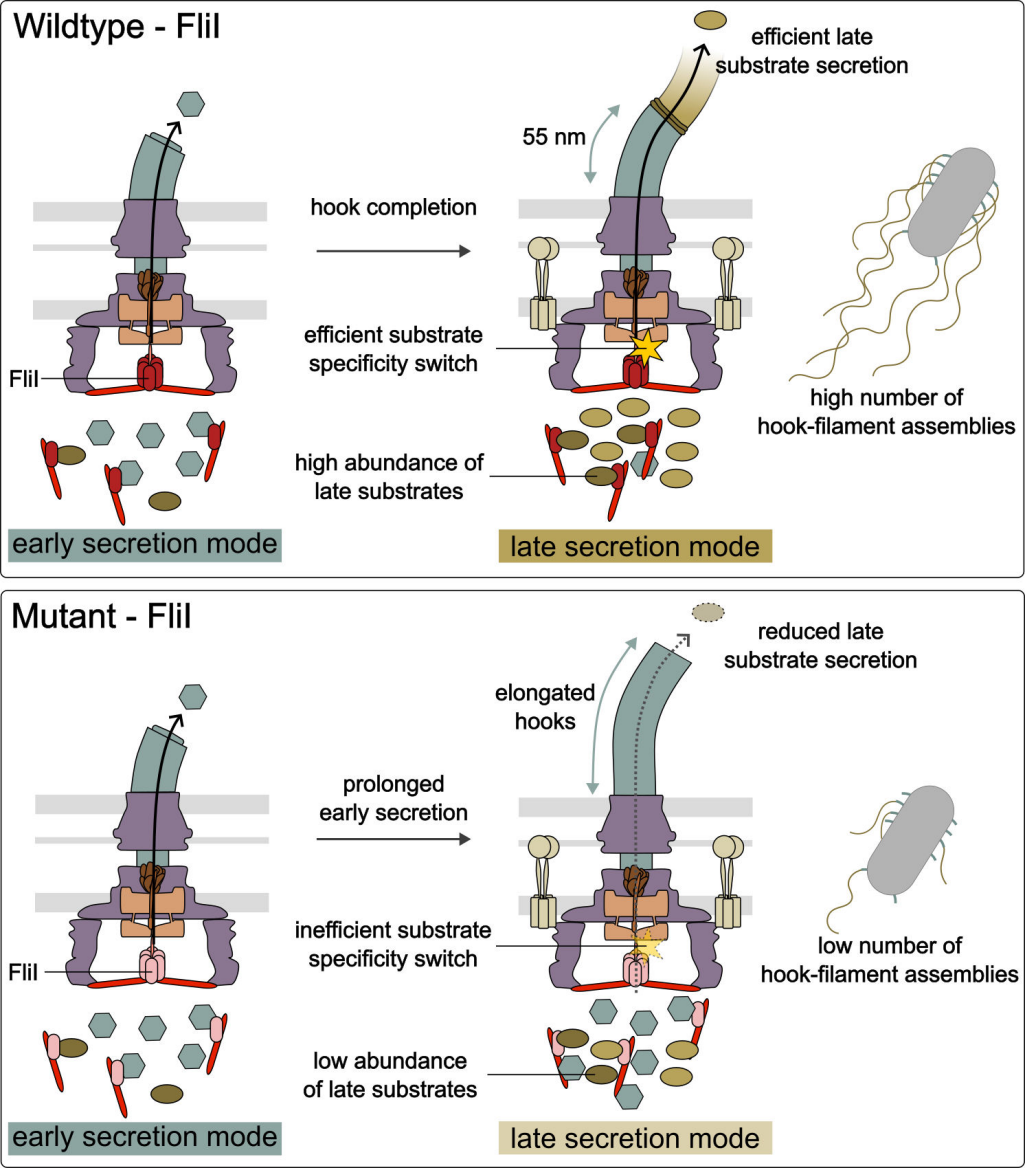
Schematic model of FliI function in WT and mutant strains. In WT cells (top), FliI facilitates the transition from early to late secretion upon hook completion. This enables efficient FlgM secretion, timely induction of class 3 gene expression, and robust export of late substrates, resulting in many hooks with attached filaments. In FliI mutants (bottom), prolonged early secretion, delayed FlgM export, impaired class 3 gene expression, and inefficient late substrate export are observed. Consequently, hooks are elongated, and fewer filaments are assembled.

The key findings are summarized in Table 1. Notably, mutants such as R177H and G183A, despite retaining early secretion and WT numbers of HBBs, exhibited strong defects in late substrate secretion and reduced gene expression from class 3 promoters (Fig. 4). All mutants showed progressively stronger defects in all late assembly metrics, despite varying ATPase activities. Importantly, these defects were not due to different FliI expression levels or loss of FliI oligomerization or localization, as demonstrated by western blotting, *in vivo* photo-crosslinking, and fluorescence microscopy.

**TABLE 1:** Characteristics of the analyzed FliI mutants

	Location/functional context	Relative motility	Relative ATPase activity	Relative early substrate secretion	Relative FliC-HiBiT secretion	Relative class 3 gene expression T_90_	Hooks per cell	Hooks with attached filament (%)	Hook length (nm)
WT		1.00	1.00	1.00	1.000	1.000	3.1	58.7	54.4 ± 4.9
∆*fliI*		0.04	NA^*a*^	0.02	0.001	0.017	NA	NA	NA
R177H	Monomer interface, FliI_CAT_	0.48	0.09	1.00	0.417	0.183	3.1	38.2	57.1 ± 6.5
G183A	Walker A motif (nucleotide binding)	0.93	0.75	1.38	0.754	0.778	3.0	50.5	58.5 ± 5.2
E211D	Walker B motif (catalysis)	0.25	0.08	0.62	0.135	0.034	2.6	24.9	NA
E211Q	Walker B motif (catalysis)	0.09	0.04	0.37	0.003	0.027	0.5	11.4	NA
E215Q	monomer interface and catalysis	0.06	0.03	0.13	0.001	0.022	0.1	0.0	NA

^*a*^ NA, not analyzed.

In addition to their defects in late substrate export, R177H and G183A substitutions caused a modest increase in hook length compared to the WT (Fig. 3D, Table 1). Interestingly, a similar phenotype has been reported for the W354A and E351/D356A substitutions in FlhA, which slightly extend the hook and inhibit secretion of late substrates (30). Furthermore, these FlhA mutations reduce the binding affinity of FlhA_C_ to FliJ, suggesting that FliJ may be required for the conformational remodeling of FlhA_C_. Because efficient FliJ-FlhA engagement is normally supported by FliH and FliI (37, 62), it is plausible that the FliI R177H and G183A variants weaken this support, despite retaining WT-level early secretion. Thus, both FlhA and FliI mutants may perturb the same remodeling pathway from opposite sides of the FliH-FliI-FliJ-FlhA axis, leading to slowed or inefficient transition into the late export state.

Consistent with this, a recent study showed that mutations in the highly conserved GYXLI motif of FlhA_C_ delayed the substrate specificity switch, resulting in polyhook phenotypes that were even more severe in the absence of FliH and FliI (31). Because these mutations did not interfere with FliJ binding, and because FlhA_L_ binds strongly to the hydrophobic dimple of FlhA_C_ to stabilize the closed state that promotes early export, it was proposed that ATP hydrolysis by the cytoplasmic ATPase complex is required to efficiently remodel FlhA_C_ to the open state, which supports late export (28, 31). Our findings are fully consistent with this model: the reduced ATPase activity in the FliI mutants may impair this remodeling step, thereby disrupting efficient switching to late substrate export. Additionally, we cannot exclude that FliI activity may contribute to dissociation of late chaperone-substrate complexes, as shown for the ATPase InvC of the evolutionarily related virulence-associated T3SS (41). Because early substrates are exported without chaperones, late-stage secretion may be more dependent on ATP hydrolysis.

Interestingly, our data also suggest that different stages of flagellar assembly place distinct demands on the ATPase activity of FliI. Mutants with strongly reduced activity (e.g., E211D, E211Q, and E215Q) were still able to support some early substrate secretion and HBB formation but failed to progress to late substrate export. Moreover, even small differences in activity among these low-activity mutants were sufficient to influence the efficiency of early substrate secretion, whereas efficient switching to late substrate export clearly required ATPase activity close to WT levels. This observation helps explain why G183A, which retains ~75% of WT activity, and R177H, which only retains 9% of WT activity, are not affected in early assembly but still impaired in late substrate export. Thus, FliI ATPase activity appears to contribute in a graded manner, with higher activity becoming essential for the conformational remodeling events that enable the substrate specificity switch and late substrate export.

A limitation of our study is that reduced secretion of late substrates cannot be completely uncoupled from the switching mechanism, since late substrate export is a direct consequence of the switch. However, we note that early substrate secretion and HBB formation were fully intact in R177H and G183A, indicating that the secretion system is functional and not globally impaired. The elongated hook phenotypes observed in these mutants, therefore, cannot be explained solely by reduced overall secretion efficiency but are more consistent with a defect or delay in the substrate specificity switch. We therefore interpret the observed phenotypes as evidence that FliI ATPase activity supports the timely remodeling of the export gate, while acknowledging that further mechanistic studies are needed to dissect whether the involvement of FliI in the remodeling of the fT3SS and in the translocation of late substrates is two distinct functions or interconnected facets of the same process.

Together, our data suggest that the ATPase activity of FliI not only energizes substrate translocation but also contributes to the conformational remodeling of the export gate that enables the transition to late substrate export. Our findings, therefore, support a model in which FliI contributes in multiple, possibly interrelated ways to flagellar assembly. First, FliI acts as a dynamic substrate carrier, delivering substrates to the export gate (40). Second, in its anchored state at the BB, its ATPase activity contributes to the activation of the proton motive force-driven export gate and promotes conformational remodeling of the fT3SS upon hook completion (31, 42). Hence, rather than serving solely as an energy-coupling translocase, FliI operates as a multi-functional ATPase that integrates substrate delivery, energy coupling, and specificity switching, underscoring its central role in flagellar biogenesis.

Our findings have broader implications for understanding how ATPases coordinate energy transduction with regulatory processes in T3SSs. By linking FliI ATPase activity to the timing and efficiency of the substrate specificity switch, this study highlights the coupling between catalytic activity and export control within the flagellar T3SS. Although analogous switching mechanisms have not been demonstrated for virulence-associated T3SSs, the structural and functional conservation of their ATPases suggests that similar principles may apply. Thus, dissecting how FliI activity contributes to temporal control of secretion provides a framework for exploring how related ATPases regulate sequential substrate export across diverse bacterial secretion systems.

## MATERIALS AND METHODS

### Bacterial strains, plasmids, and media

All bacterial strains and plasmids used in this study are listed in Table S2 and were derived from *S. enterica* subsp. *enterica* serovar Typhimurium LT2. Cells were cultivated at 37°C under aerobic conditions in lysogeny broth (LB) at 180 rpm. If not stated otherwise, the main cultures were diluted 1:100 and grown to mid-exponential phase. If required, cultures were supplemented with ampicillin (cf = 100 µg/mL), chloramphenicol (Cm; cf = 12.5 or 10 µg/mL), and/or AnTc (cf = 100 ng/mL). The FliI point mutations were introduced chromosomally by site-specific mutagenesis using λ-Red homologous recombination (63). For each experiment, mutants and the corresponding WT control were generated in the same parental background to maintain isogenicity; thus, the only differences between WT and mutant strains within a given experiment are the defined point mutations in *fliI*. Oligonucleotides used in this study are listed in Table S3. Furthermore, the generalized transducing phage of *Salmonella enterica* serovar Typhimurium P22 HT105/1 int-201 was used (64). Plasmids were constructed using NEBuilder HiFi DNA Assembly Master Mix (New England Biolabs, catalog number E2621L) or one-step mutagenesis (65).

### Swimming motility assay

Swimming motility was studied using tryptone broth-based soft agar swim plates containing 0.3% Bacto agar. Motility plates were inoculated with 2 µL of overnight culture and incubated at 37°C for 4–5 h. Images were acquired by scanning the plates, and the diameters of the swimming halos were measured using Fiji (66). The swimming diameters of the mutant strains were normalized to those of the WT.

### Quantification of cellular FliI levels

Strains expressing FLAG-tagged FliI variants were grown until the late-exponential phase. OD_600_ was measured, and 1 mL of aliquot was taken. Cells were harvested by centrifugation at 13,000 × *g* for 5 min. The cell pellets were resuspended in 1 mL of double-distilled water. Proteins were precipitated using 10% (vol/vol) trichloroacetic acid (TCA), followed by 30 min incubation on ice. Proteins were pelleted by centrifugation at 20,000 × *g* for 30 min at 4°C. Protein pellets were washed with ice-cold acetone and air dried. Samples were adjusted to 20 OD units/µL. A total of 200 OD units were analyzed under denaturing conditions using SDS-PAGE (12% Mini-PROTEAN TGX Stain-Free Protein Gels). Total protein detection was carried out to allow normalization of FliI levels. Immunoblotting was performed using primary α-FLAG antibody (Sigma-Aldrich, catalog number F1804-200UG, 1:10,000 in 1× Tris-buffered saline with Tween 20 [TBS-T]). Proteins were detected using secondary α-mouse antibodies conjugated to horseradish peroxidase (Bio-Rad Immun-Star Goat Anti-Mouse [GAM]-horseradish peroxidase [HRP] Conjugate, catalog number 170-5047, 1:20,000 in 1× TBS-T). The relative amounts of cellular proteins were determined by normalization to the total protein using the Image Lab software (Bio-Rad).

### ATPase activity measurements

His_6_-SUMO-tagged FliI and FliI variants were expressed in *S. enterica* from pSUMO under the T7 promoter in strains carrying a chromosomal T7 RNA polymerase gene under P*_lac_* control (67). Cultures were grown overnight in LB medium supplemented with kanamycin, diluted 1:100 into 500 mL LB, and incubated at 37°C until reaching an OD_600_ of 0.5–0.7. Protein expression was induced with 0.2 mM isopropyl β-D-1-thiogalactopyranoside (IPTG), and cells were further incubated at 30°C for 3 h before harvesting at 10,000 × *g* for 15 min at 4°C. Cell pellets were frozen at −20°C until further use.

The purification protocol was adapted from Fan and Macnab (68). Briefly, cell pellets were resuspended in binding buffer (20 mM Tris-HCl pH 8.0, 500 mM NaCl, 1 mM β-mercaptoethanol, and 5 mM imidazole) and lysed by sonication on ice. After removal of cell debris at 17,000 × *g* for 40 min, the SN was clarified by ultracentrifugation at 150,000 × *g* for 2.5 h. The soluble fraction was incubated overnight with Ni-NTA agarose (Qiagen) pre-equilibrated in binding buffer. Beads were washed sequentially with binding buffer containing 5 and 25 mM imidazole, and bound proteins were eluted with a stepwise imidazole gradient (50–250 mM). Pure fractions containing FliI were pooled, and protein concentrations were determined using the Bradford assay (Bio-Rad).

The His_6_-SUMO tag was cleaved during dialysis against dialysis buffer (20 mM Tris-HCl pH 8.0, 50 mM NaCl, 0.25 mM EDTA, and 1 mM β-mercaptoethanol) using His_6_-ULP1 protease (1:20 [wt/wt] ratio of His_6_-ULP1:substrate). Dialysis was performed with two buffer exchanges (2 h and overnight) at 4°C. Cleaved proteins were separated from the His_6_-SUMO tag and His_6_-ULP1 by re-binding to Ni-NTA resin, collecting the flowthrough. Protein purity was assessed by SDS-PAGE and Coomassie staining.

ATPase activity of purified FliI variants was determined using the malachite green assay adapted from Lanzetta et al. (69). Briefly, 100 µL reactions containing 1 µM protein in assay buffer (50 mM Tris-HCl pH 7.8, 150 mM NaCl, 5 mM MgCl_2_, 2 mM dithiothreitol [DTT]) were incubated at 37°C and initiated by the addition of 4 mM ATP. At defined time points (0–30 min), 10 µL aliquots were removed and immediately quenched in 160 µL freshly prepared malachite green-molybdate reagent (0.045% [wt/vol] malachite green hydrochloride, 1% [wt/vol] ammonium molybdate in 1 M HCl, and 0.1% [vol/vol] Triton X-100) in a 96-well plate. The reaction was stopped by adding 20 µL of 40% sodium citrate. Inorganic phosphate was quantified at 640 nm using a Tecan microplate reader against a standard curve of KH_2_PO_4_ (0–1 mM). At least three independent measurements were carried out, and negative controls without ATP and protein were included to exclude contaminating phosphate. ATPase activities were normalized to the FliI WT variant.

### Subcellular localization of FliI

The strains carried translational fusions of *fliG* to mNeonGreen and of HaloTag to *fliI*. Additionally, the strains carried an AnTc inducible P*_tetA_* promoter for the flagellar master regulator operon *flhDC* (44). Overnight cultures were grown in 2 mL LB. Cultures were diluted 1:100 in 10 mL of fresh LB and incubated at 37°C for 1.5 h. The expression of *flhDC* was induced by the addition of AnTc (cf = 100 ng/µL), followed by 1 h incubation at 37°C. A 500 µL sample of each culture was collected and centrifuged at 2,550 × *g* for 3 min. Pellets were resuspended in 1× phosphate buffered saline (PBS) and adjusted to an OD_600_ of 0.3. A 150 µL sample of each strain was transferred to a fresh 1.5 mL reaction tube, and HaloTag labeling was performed by adding the HaloTag ligand TMR (cf = 0.5 µM, Promega, catalog number G8251) and incubating the samples for 30 min at 37°C. Cells were washed twice with 500 µL of 1× PBS and resuspended in 300 µL of 1× PBS for microscopy. For imaging, 1 µL of labeled cells was spotted onto a 1% agarose pad in 1× PBS. The cell suspension spot was air dried for approximately 1 min, and plasma-cleaned glass coverslips were applied. Fluorescence microscopy was performed using a Ti-2 Nikon inverted microscope equipped with a CFI Plan Apochromat DM 60× Lambda oil Ph3/1.40 (Nikon) oil objective, an Orca Fusion BT camera (Hamamatsu), and a SPECTRA III LED light source (Lumencor). *Z*-stacks were acquired in a 1.6 µm range with five stacks at 0.4 µm intervals. Microscopy data were segmented using Ilastik v1.4.0 (70) and analyzed using a custom Jupyter Notebook (see data availability statement).

### *In vivo* photo-crosslinking of the FliI hexamer

The protocol for *in vivo* photo-crosslinking to study the ability of FliI to form hexamers was adapted from previously published protocols (71, 72). Strains were deleted for *fliI*, carried pSUP, and expressed FLAG-tagged FliI (with amber mutation and respective point mutations) constitutively from pTrc99A-FF4. Overnight cultures were grown in LB, supplemented with Cm (cf = 10 µg/mL) and ampicillin (cf = 100 µg/mL) at 37°C, 180 rpm. Main cultures were diluted 1:100 in 10 mL LB with the respective supplements in the presence of 1 mM *p*Bpa at 37°C until they reached mid-exponential phase. Nine milliliters of each culture was harvested at 4,000 × *g*, 4°C for 5 min. Cells were washed once with 2 mL cold 1× PBS and pelleted again with centrifugation. Cells were resuspended in 2 mL cold 1× PBS. One milliliter of the cell culture was transferred into a fresh 1.5 mL reaction tube; another 1 mL was transferred into a six-well cell culture dish and irradiated for 30 min with UV light (λ = 366 nm) on ice. Afterward, the cells were transferred into a fresh 1.5 mL reaction tube and harvested at 10,000 × *g* at 4°C for 3 min. Proteins were precipitated as described above. Samples were adjusted to 50 OD units/µL. A total of 500 OD units were analyzed under denaturing conditions using SDS-PAGE. Immunoblotting was performed using primary α-FLAG antibody (Sigma-Aldrich, catalog number F1804-200UG, 1:10,000 in 1× TBS-T). Proteins were detected using secondary α-mouse antibodies conjugated to horseradish peroxidase (Bio-Rad Immun-Star GAM-HRP Conjugate, catalog number 170-5047, 1:20,000 in 1× TBS-T).

### Minimal inhibitory concentration assay (FlgE-Bla secretion)

The minimal inhibitory concentration of ampicillin correlating with the amount of secreted hook-protein-β-lactamase fusion protein (FlgE-Bla) was determined as described by Lee et al. (47) and adapted by Singer et al. (48) to fit a 96-well plate format.

Briefly, cultures were grown overnight in 200 µL LB in a 96-well plate at 37°C, 180 rpm in biological triplicate. The cultures were diluted 1:100 in fresh LB and grown to mid-exponential phase at 37°C at 180 rpm. The cultures were then diluted 1:10 in 300 µL LB and finally diluted 1:10 in 200 µL LB media supplemented with increasing concentrations of fresh ampicillin (Amp) in technical triplicates. Cells were grown for 4.5 h at 37°C at 180 rpm, and OD_600_ was measured with a microplate reader. The IC_50_ was calculated using a custom Jupyter Notebook.

### Secretion assay using the split NanoBit enzyme

Nano-Glo HiBiT Extracellular Detection System was purchased from Promega (N2420). The quantification of the secreted HiBiT-tagged proteins was determined as described previously (52). Briefly, overnight cultures were diluted 1:100 and grown to the late exponential phase at 37°C at 180 rpm. OD_600_ was measured, and a 1 mL sample of each culture was transferred into a 1.5 mL Eppendorf tube on ice. Samples were centrifuged at 13,000 × *g* at 4°C for 3 min. A total of 500 µL of the SN was transferred into a fresh Eppendorf tube and stored on ice. A total of 25 µL of the SN was transferred in technical triplicate into a white 384-well plate with LB-blanks included. The working solution was prepared, and the SN samples and the working solution were mixed as described by the manufacturer. Luminescence was measured at *t*_0_ = 0 min and *t*_10_ = 10 min using a microplate reader. The relative light units (RLU) of each strain were calculated using the following equation:


$$RLU=\left(t_{10}-t_{0}\right)-\left(mean\;of\;blank\right)$$

The calculated values were normalized to the measured OD_600_ and to the WT strain.

### Secretion assay analyzed by SDS-PAGE and western blot

For analysis with SDS-PAGE and western blotting, the same cultures were used as for the secretion assay using the split luciferase. A 1.9 mL aliquot was taken, and cells were harvested by centrifugation at 13,000 × *g* for 5 min. One milliliter of the SN was transferred into a fresh tube, the remaining SN was discarded, and the cell pellets were resuspended in 1 mL of double-distilled water. Proteins were precipitated as described above. Samples were adjusted to 20 OD units/µL. A total of 200 OD units were analyzed under denaturing conditions using SDS-PAGE. The samples of all three biological replicates were pooled to obtain the mean protein amount of all replicates. Immunoblotting was performed using primary α-FlgM (gift from K. Hughes, 1:10,000 in 1× TBS-T), α-FlgK (gift from T. Minamino, 1:10,000 in 1× TBS-T), α-FliD (gift from T. Minamino, 1:10,000 in 1× TBS-T), or α-FliC (Difco, catalog number 228241 *Salmonella* H Antiserum I, 1:5,000 in 1× TBS-T) antibodies. Proteins were detected using secondary α-rabbit antibodies conjugated to horseradish peroxidase (Bio-Rad Immun-Star Goat Anti-Rabbit-HRP Conjugate, catalog number 170-5046, 1:20,000 in 1× TBS-T). The relative amounts of secreted and cellular proteins were determined by normalization to the housekeeping protein DnaK using the Image Lab software (Bio-Rad). DnaK was detected using a primary α-DnaK (abcam, catalog number ab69617, 1:10,000 in 1× TBS-T) antibody and secondary α-mouse antibodies conjugated to horseradish peroxidase (Bio-Rad Immun-Star GAM-HRP Conjugate, catalog number 170-5047, 1:20,000 in 1× TBS-T).

### Luciferase assay - P*_motA_***-***luxCDABE*

Strains harboring the plasmid pRG19 (P*_motA_-luxCDABE*) were used to monitor flagellar class 3 gene expression (58). ON-cultures of six biological replicates were grown in 200 µL LB supplemented with Cm with cf = 12.5 µg/mL in a 96-well plate at 37°C, 200 rpm. To prevent evaporation, the plate was covered with a Breath-Easy sealing membrane. The cultures were diluted 1:10 in 200 µL LB supplemented with Cm and again 1:10 in LB supplemented with Cm and AnTc to a final concentration of 100 ng/mL to induce expression of the flagellar master regulator from P*_tetA_* (44) in a white 96-well plate for final dilution to 1:100. The OD_600_ was measured, as well as luminescence with a microplate reader for 6 h every 15 min. The RLUs were normalized to the measured OD_600_ of each sample and analyzed as described previously (59, 73). The relative class 3 gene expression in respect to the WT was calculated for three timepoints *t*_360_ (360 min).

### Hook and filament staining

For determination of the number of hooks per cell and the number of hooks with an attached filament, cells were grown to mid-log phase. A total of 500 µL samples were taken and centrifuged at 2,500 × *g* for 5 min. The cells were carefully resuspended in 500 µL fresh 1× PBS. Star Green maleimide dye (Abberior, catalog number STGREEN-0003-1MG) with cf = 10 µM and 0.5 µL α-HA-Alexa555 (Thermo Fisher Scientific, catalog number 26183-A555) were added to stain the filaments (*fliC*_T237C_) (74) with maleimide and the hook (*flgE*_3×HA_) with immunostaining. The cells were incubated for 1 h at 37°C at 300 rpm. The cells were washed twice with 1× PBS before loading the samples on a poly-L-lysine coated coverslip as described previously (75, 76). The cells were fixed using 2% paraformaldehyde-0.2% glutaraldehyde solution and rinsed with 1× PBS before staining the DNA fluoroshield containing 4′,6-diamidino-2-phenylindole (DAPI, Sigma Aldrich, catalog number F6057-20ML). Cells were observed using a Zeiss Inverted Microscope Axio Observer Z1 at 100× magnification, and *z*-stack experiments were performed with nine slices, 4 µm range with 0.5 µm slice interval. Analysis was done using Fiji for image processing, equipped with the MicrobeJ Plugin to determine the number of hooks per cell (66, 77). Filaments were counted manually on cells with determined hook numbers.

### Hook length measurements

The purification of HBB complexes was adapted from a previously published protocol (6, 16, 78). Briefly, an overnight culture was inoculated with a single colony in LB medium. The next day, 500 mL LB medium was inoculated 1:100 with the overnight culture and grown for 2.5 h. We utilized strains with an AnTc-inducible P*_tetA_* promoter for the flagellar master regulator operon *flhDC* (44). To induce flagellar biosynthesis, AnTc was added, and the cells were further inoculated for 1 h. Cells were harvested at 4,000 × *g* at 4°C for 15 min. The cell pellet was carefully resuspended in 20 mL ice-cold sucrose solution (0.5 M sucrose, 0.15 M Trizma base, and unaltered pH) on ice. Lysozyme and EDTA pH 4.7 were added slowly to final concentrations of 0.1 mg/mL and 2 mM, respectively, while stirring the cell suspension on ice. After 5 min of stirring on ice, the suspension was transferred to room temperature (RT) and slowly stirred for 1 h to allow spheroplast formation. For cell lysis, Triton X-100 was added to a final concentration of 1%. After rapidly stirring the suspension for 10 min until it became translucent, it was slowly stirred further for 30–45 min on ice. To degrade DNA, 2 mg DNase I and MgSO_4_ were added to a final concentration of 5 mM while stirring on ice. After 5 min, EDTA pH 4.7 was added at a final concentration of 5 mM. To pellet cell debris and unlysed cells, the suspension was centrifuged at 15,000 × *g* at 4°C for 10 min. The SN was collected and centrifuged at 37,000 rpm (T-647.5 Fixed Angle Rotor, Thermo Fisher Scientific) for 1 h at 4°C to pellet the flagella. The flagella were washed carefully with 50 mL buffer A (0.1 M KCl, 0.3 M sucrose, and 0.1% Triton X-100) and collected again at 37,000 rpm (T-647.5 Fixed Angle Rotor, Thermo Fisher Scientific) for 1 h at 4°C. To depolymerize the flagellin filament, the pellet was carefully resuspended in 50 mL buffer B (50 mM glycine, 0.1% Triton X-100, pH 2.5), followed by 30 min incubation at room temperature. Finally, the HBBs were collected again by centrifugation at 37,000 rpm (T-647.5 Fixed Angle Rotor, Thermo Fisher Scientific) for 1 h at 4°C. HBBs were resuspended carefully in 50 µL buffer C (10 mM Tris pH 8, 5 mM EDTA pH 8, and 0.1% Triton X-100) and incubated overnight on a rolling platform at 4°C. Grids were prepared as described previously (79). Briefly, aliquots of HBB samples were applied to freshly glow-discharged carbon-film-coated copper grids and allowed to adsorb for 10 min. After three washes with distilled water, the grids were contrasted with 4% phospho-tungstic-acid/1% trehalose, touched on filter paper, and air dried. The grids were analyzed in a Leo 906 transmission electron microscope (Zeiss) at 100 kV acceleration voltage. Micrographs were scanned using a Morada side-mount camera (SIS) with ImageSP software from Tröndle Restlichtverstärkersysteme (TRS) . Hook lengths were measured using Fiji (66).

### Statistical analyses

Statistical analyses were performed using GraphPad Prism 10 (GraphPad Software), and values of *P* < 0.05 were considered statistically significant.

## Data Availability

The Jupyter notebook used for colocalization analyses of the microscopy data is available at https://github.com/SalmoLab/FliI_ATPase.

## SUPPLEMENTAL MATERIAL

Supplemental Information mbio.02354-25-s0001.docx Fig. S1 to S4 and Tables S1 to S3.

ASM does not own the copyrights to Supplemental Material that may be linked to, or accessed through, an article. The authors have granted ASM a non-exclusive, world-wide license to publish the Supplemental Material files. Please contact the corresponding author directly for reuse.
