# Supplementary material for: The FliI ATPase couples ATP hydrolysis to substrate switching in bacterial flagellar type-III secretion
Source: mBio. 2025 Dec 5;17(1):e02354-25. doi: 10.1128/mbio.02354-25 (PMC12802181; doi:10.1128/mbio.02354-25)
Supplement: Supplemental Information — Fig. S1 to S4 and Tables S1 to S3. [file mbio.02354-25-s0001.docx]

**Supplementary information for:**

**The FliI ATPase couples ATP hydrolysis to substrate switching in bacterial flagellar type-III secretion**

Rosa Einenkel^a^, Caroline Kühne^a^, Mario Delgadillo‐Guevara^a,b^, Lasse Hallenga^a^, Christian Goosmann^c^, and Marc Erhardt^a,d^#

^a^Humboldt-Universität zu Berlin, Institute of Biology, 10115 Berlin, Germany

^b^Department of Biochemistry and Pharmacology, Bio21 Molecular Science and Biotechnology Institute, The University of Melbourne, Melbourne, VIC, Australia

^c^Max Planck Institute for Infection Biology, 10117 Berlin, Germany

^d^Max Planck Unit for the Science of Pathogens, 10117 Berlin, Germany

Running Head: FliI ATPase Controls Flagellar Secretion Specificity

#Correspondence to: [rosa.einenkel@hu-berlin.de](mailto:rosa.einenkel@hu-berlin.de) or [marc.erhardt@hu-berlin.de](mailto:marc.erhardt@hu-berlin.de)

**Table of Contents**

Figure S1. Localization and oligomerization of FliI and FliI mutants.

Figure S2. FliI is required for efficient secretion of late flagellar substrates.

Figure S3. Quantification of class 3 gene expression.

Figure S4. FliI mutants exhibit a decreased number of hooks with attached filaments.

Table S1. Statistics of the colocalization analysis.

Table S2. Strains and plasmids used in this study.

Table S3. Oligonucleotides used in this study.

Supplementary References

**Fig. S1**. Localization and oligomerization of FliI and FliI mutants. (A) Distance Distribution Plots from the colocalization analysis of FliI-FliG colocalization. Violin plots show data distributions of each dataset and black horizontal line represents the median. (B) Representative microscopy images of FliI-HaloTag in various genetic backgrounds. FliI-HaloTag labeled with TMR-ligand (magenta), basal bodies visualized with mNeonGreen-FliG (cyan); PC (PhaseContrast). Scale-bar: 2 µm. (C) Schematic of the *in vivo* site-specific photo-crosslinking system used to assess FliI oligomerization. An amber codon is introduced at position D341 of FliI to incorporate the photo-reactive amino acid p-benzoyl-L-phenylalanine (pBpa). Upon UV irradiation, crosslinking occurs between interacting FliI subunits. (D) *In vivo* UV crosslinking of FLAG-tagged FliI^D341pBpa^ variants expressed constitutively from pTrc99A-FF4 in a ∆*fliI* background. High molecular weight (HMW) adducts were detected for WT and all point mutants, confirming oligomerization. Samples were separated by SDS-PAGE and visualized by immunoblotting against FLAG.

**Fig. S2**. FliI is required for efficient secretion of late flagellar substrates. (A) Quantification of early substrate (FlgE-Bla) secretion. Shown are IC₅₀ values for ampicillin resistance, reflecting the amount of secreted FlgE-Bla. Bars represent means ± SD with individual data points shown from ≥3 independent experiments. Statistical significance was determined by one-way ANOVA with Dunnett’s multiple comparisons test vs. Δ*fliF.* ***p < 0.001; **p < 0.01; *p < 0.05; ns, not significant. (B-E) Western Blot analysis of FlgM-HiBiT (B), FlgK-HiBiT (C), FliD-HiBiT (D) and FliC-HiBiT in cellular and secrete fractions. Samples were pooled from 3 independent experiments, separated by SDS-PAGE and immunoblotted using anti-DnaK and anti-FlgM, anti-FlgK, anti-FliD or anti-FliC, respectively. Numbers indicate the amount of HiBiT-tagged proteins normalized to DnaK and to the WT.

**Fig. S3**. Quantification of class 3 gene expression. Relative class 3 gene expression (P*_motA_* activity) at 90 min (T_90_) and 360 min (T_360_) after induction of flagellar synthesis. Shown are the normalized luminescence values from six biological replicates, expressed relative to the WT signal at each time point. Bars represent means ± SD with individual data points shown.

**Fig. S4**. FliI mutants exhibit a decreased number of hooks with attached filaments. Shown are the numbers of filaments per cell as a function of hook number per cell. Cells were FliC^ON^ and expressed *flgE*_3×HA_ and *fliC*_T237C_ for immuno- and maleimide staining of the hook and filament, respectively. The width of the violin plot outline illustrates the distribution of the data. The numbers above each violin bar represent the number of counted cells for this category. Thick dashed lines represent the median; dotted lines represent the quartiles.

**Table S1.** Statistics of the colocalization analysis.

|  | **WT** | **∆2-7** | **R177H** | **G183A** | **E211D** | **E211Q** | **E215Q** |
| --- | --- | --- | --- | --- | --- | --- | --- |
| **Cells total** | 428 | 1489 | 1638 | 1207 | 655 | 1377 | 1662 |
| **Total pairs** | 1042 | 971 | 3778 | 2723 | 1560 | 3212 | 4139 |
| **Total pairs/cell** | 2,4 | 0,7 | 2,3 | 2,3 | 2,4 | 2,3 | 2,5 |
| **Valid pairs** | 788 | 157 | 2722 | 1923 | 1146 | 2327 | 2910 |
| **Valid pairs / cell** | 1,8 | 0,1 | 1,7 | 1,6 | 1,8 | 1,7 | 1,8 |

**Table S2.** Strains and Plasmids used in this study.

| **Strain** | **Genotype** | **Source/Reference** |
| --- | --- | --- |
| EM2624 | LT2 wild-type | Lab Collection |
| TH12424 | ∆*fliI*7364 | Lab Collection |
| **EM4120** | *fliI*23023 (R177H) | Lab Collection |
| EM4121 | *fliI*23024 (G183A) | Lab Collection |
| EM9307 | *fliI*23209 (E211D) | Lab Collection |
| EM9308 | *fliI*23210 (E211Q) | Lab Collection |
| EM4122 | *fliI*23025 (E215Q) | Lab Collection |
| EM9623 | trp::[Spc^R^ T7-RNAP *lacO* *lacP* *lacI*] / pEM9411 (pSUMO-*fliI* (full length), Kan^R^) | This study |
| EM11139 | trp::[Spc^R^ T7-RNAP *lacO* *lacP* *lacI*] / pEM11119 (pSUMO-*fliI* (full length, R177H), Kan^R^) | This study |
| EM11140 | trp::[Spc^R^ T7-RNAP *lacO* *lacP* *lacI*] / pEM11120 (pSUMO-*fliI* (full length, G183A), Kan^R^) | This study |
| EM11141 | trp::[Spc^R^ T7-RNAP *lacO* *lacP* *lacI*] / pEM11121 (pSUMO-*fliI* (full length, E211D), Kan^R^) | This study |
| EM11142 | trp::[Spc^R^ T7-RNAP *lacO* *lacP* *lacI*] / pEM11122 (pSUMO-*fliI* (full length, E211Q), Kan^R^) | This study |
| EM11146 | trp::[Spc^R^ T7-RNAP *lacO* *lacP* *lacI*] / pEM11144 (pSUMO-*fliI* (full length, E215Q), Kan^R^) | This study |
| EM13539 | *fliI*23539-SAGASA-3×FLAG (C-ter) | This study |
| EM15150 | *fliI*23682(∆aa2-7)-SAGASA-3×FLAG (C-ter) | This study |
| EM15151 | *fliI*23683(R177H)-SAGASA-3×FLAG (C-ter) | This study |
| EM15152 | *fliI*23684(G183A)-SAGASA-3×FLAG (C-ter) | This study |
| EM15153 | *fliI*23685(E211D)-SAGASA-3×FLAG (C-ter) | This study |
| EM15154 | *fliI*23686(E211Q)-SAGASA-3×FLAG (C-ter) | This study |
| EM15155 | *fliI*23687(E215Q)-SAGASA-3×FLAG (C-ter) | This study |
| EM14153 | *fliI*23109 (*fliI*-HaloTag) *fliG*22799 (mNeonGreen-*fliG*) P*_flhDC_*5451::Tn10dTc[del-25] | This study |
| EM14391 | *fliG*22799 (mNeonGreen-*fliG*) P*_flhDC_*5451::Tn10dTc[del-25] *fliI*23619 (R177H)::HaloTag (C-term) | This study |
| EM15105 | *fliG*22799 (mNeonGreen-*fliG*) P*_flhDC_*5451::Tn10dTc[del-25] *fliI*236677 (G183A)::HaloTag (C-term) | This study |
| EM14392 | *fliG*22799 (mNeonGreen-*fliG*) P*_flhDC_*5451::Tn10dTc[del-25] *fliI*23620 (E211D)::HaloTag (C-term) | This study |
| EM14393 | *fliG*22799 (mNeonGreen-*fliG*) P*_flhDC_*5451::Tn10dTc[del-25] *fliI*23621 (E211Q)::HaloTag (C-term) | This study |
| EM14394 | *fliG*22799 (mNeonGreen-*fliG*) P*_flhDC_*5451::Tn10dTc[del-25] *fliI*23622 (E215Q)::HaloTag (C-term) | This study |
| EM15149 | *fliG*22799 (mNeonGreen-*fliG*) P*_flhDC_*5451::Tn10dTc[del-25] *fliI*23681(∆aa2-7)::HaloTag (C-term) | This study |
| EM14389 | *fliI*23109 (*fliI*-HaloTag) *fliG*22799 (mNeonGreen-*fliG*) P*_flhDC_*5451::Tn10dTc[del-25] ∆*fliH*7363 | This study |
| EM14390 | *fliI*23109 (*fliI*-HaloTag) *fliG*22799 (mNeonGreen-*fliG*) P*_flhDC_*5451::Tn10dTc[del-25] ∆*fliJ*7365 | This study |
| EM15298 | *fliI*23109 (*fliI*-HaloTag) *fliG*22799 (mNeonGreen-*fliG*) P*_flhDC_*5451::Tn10dTc[del-25] ∆*fliN*23676::FRT | This study |
| EM15299 | *fliI*23109 (*fliI*-HaloTag) *fliG*22799 (mNeonGreen-*fliG*) P*_flhDC_*5451::Tn10dTc[del-25] ∆*flhA*23464::FRT | This study |
| EM14428 | *fliI*23109 (*fliI*-HaloTag) *fliG*22799 (mNeonGreen-*fliG*) P*_flhDC_*5451::Tn10dTc[del-25] ∆*flgBC*23587::FRT | This study |
| EM14429 | *fliI*23109 (*fliI*-HaloTag) *fliG*22799 (mNeonGreen-*fliG*) P*_flhDC_*5451::Tn10dTc[del-25] ∆*flgE*22964::FRT | This study |
| EM14430 | *fliI*23109 (*fliI*-HaloTag) *fliG*22799 (mNeonGreen-*fliG*) P*_flhDC_*5451::Tn10dTc[del-25] ∆*flgKL*5739::FRT | This study |
| EM14456 | ∆*fliI*7364 / pEM14338 (pTrc99A-FF4-*fliI* (D341amber)-SAGASA-3×FLAG, C-ter, Amp^R^) / pSUP (Cm^R^) | This study |
| EM15252 | ∆*fliI*7364 / pEM15178 (pTrc99A-FF4-*fliI* (∆aa2-7, D341amber)-SAGASA-3×FLAG, C-ter, Amp^R^) / pSUP (artificial amino-acid, Cm^R^) | This study |
| EM15253 | ∆*fliI*7364 / pEM15179 (pTrc99A-FF4-*fliI* (R177H, D341amber)-SAGASA-3×FLAG, C-ter, Amp^R^) / pSUP (artificial amino-acid, Cm^R^) | This study |
| EM15254 | ∆*fliI*7364 / pEM15180 (pTrc99A-FF4-*fliI* (G183A, D341amber)-SAGASA-3×FLAG, C-ter, Amp^R^) / pSUP (artificial amino-acid, Cm^R^) | This study |
| EM15255 | ∆*fliI*7364 / pEM15181 (pTrc99A-FF4-*fliI* (E211D, D341amber)-SAGASA-3×FLAG, C-ter, Amp^R^) / pSUP (artificial amino-acid, Cm^R^) | This study |
| EM15256 | ∆*fliI*7364 / pEM15182 (pTrc99A-FF4-*fliI* (E211Q, D341amber)-SAGASA-3×FLAG, C-ter, Amp^R^) / pSUP (artificial amino-acid, Cm^R^) | This study |
| EM15257 | ∆*fliI*7364 / pEM15183 (pTrc99A-FF4-*fliI* (E215Q, D341amber)-SAGASA-3×FLAG, C-ter, Amp^R^) / pSUP (artificial amino-acid, Cm^R^) | This study |
| TH9949 | *flgE*6569::*bla* ∆*flgBC*6557 (Amp^R^) | (1) |
| TH12465 | *flgE*6569::*bla* ∆*flgBC*6557 ∆*fliF*7387 | (2) |
| TH12473 | *flgE*6569::*bla* ∆*flgBC*6557 ∆*fliI*7395 | (2) |
| EM7801 | *flgE*6569::*bla* ∆*flgBC*6557 *fliI*23023 (R177H) | Lab Collection |
| EM7802 | *flgE*6569::*bla* ∆*flgBC*6557 *fliI*23024 (G183A) | Lab Collection |
| EM7803 | *flgE*6569::*bla* ∆*flgBC*6557 *fliI*23025 (E215Q) | Lab Collection |
| EM9432 | *flgE*6569::*bla* ∆*flgBC*6557 *fliI*23209 (E211D) | Lab Collection |
| EM9433 | *flgE*6569::*bla* ∆*flgBC*6557 *fliI*23210 (E211Q) | Lab Collection |
| EM9743 | *flgM*23271::HiBiT (C-ter, RBS FlgN duplicated) | Lab Collection |
| EM10103 | *flgM*23271::HiBiT (C-ter, RBS FlgN duplicated) ∆*fliI*7364 | This study |
| EM10104 | *flgM*23271::HiBiT (C-ter, RBS FlgN duplicated) *fliI*23023 (R177H) | This study |
| EM10105 | *flgM*23271::HiBiT (C-ter, RBS FlgN duplicated) *fliI*23024 (G183A) | This study |
| EM10107 | *flgM*23271::HiBiT (C-ter, RBS FlgN duplicated) *fliI*23209 (E211D) | This study |
| EM10108 | *flgM*23271::HiBiT (C-ter, RBS FlgN duplicated) *fliI*23210 (E211Q) | This study |
| EM18068 | *flgM*23271::HiBiT (C-ter, RBS FlgN duplicated) *fliI*23025 (E215Q) | This study |
| EM9999 | ∆*hin*-5717::FRT *flgK*23312::HiBiT (after aa442, 3×SAGASA-HiBiT-3×SAGASA) | Lab Collection |
| EM18069 | ∆*hin*-5717::FRT *flgK*23312::HiBiT (after aa442, 3×SAGASA-HiBiT-3×SAGASA) ∆*fliI*7364 | This study |
| EM18070 | ∆*hin*-5717::FRT *flgK*23312::HiBiT (after aa442, 3×SAGASA-HiBiT-3×SAGASA) *fliI*23023 (R177H) | This study |
| EM18071 | ∆*hin*-5717::FRT *flgK*23312::HiBiT (after aa442, 3×SAGASA-HiBiT-3×SAGASA) *fliI*23024 (G183A) | This study |
| EM18072 | ∆*hin*-5717::FRT *flgK*23312::HiBiT (after aa442, 3×SAGASA-HiBiT-3×SAGASA) *fliI*23209 (E211D) | This study |
| EM18073 | ∆*hin*-5717::FRT *flgK*23312::HiBiT (after aa442, 3×SAGASA-HiBiT-3×SAGASA) *fliI*23210 (E211Q) | This study |
| EM18074 | ∆*hin*-5717::FRT *flgK*23312::HiBiT (after aa442, 3×SAGASA-HiBiT-3×SAGASA) *fliI*23025 (E215Q) | This study |
| EM18085 | ∆*hin*-5717::FRT *fliD*23314::HiBiT (∆aa205-209, 3×SAGASA-HiBiT-3×SAGASA) ∆*flgKL*5739::FRT | This study |
| EM18174 | ∆*hin*-5717::FRT *fliD*23314::HiBiT (∆aa205-209, 3×SAGASA-HiBiT-3×SAGASA) ∆*flgKL*5739::FRT ∆*fliI*7364 | This study |
| EM18175 | ∆*hin*-5717::FRT *fliD*23314::HiBiT (∆aa205-209, 3×SAGASA-HiBiT-3×SAGASA) ∆*flgKL*5739::FRT *fliI*23023 (R177H) | This study |
| EM18176 | ∆*hin*-5717::FRT *fliD*23314::HiBiT (∆aa205-209, 3×SAGASA-HiBiT-3×SAGASA) ∆*flgKL*5739::FRT *fliI*23024 (G183A) | This study |
| EM18177 | ∆*hin*-5717::FRT *fliD*23314::HiBiT (∆aa205-209, 3×SAGASA-HiBiT-3×SAGASA) ∆*flgKL*5739::FRT *fliI*23209 (E211D) | This study |
| EM18178 | ∆*hin*-5717::FRT *fliD*23314::HiBiT (∆aa205-209, 3×SAGASA-HiBiT-3×SAGASA) ∆*flgKL*5739::FRT *fliI*23210 (E211Q) | This study |
| EM18179 | ∆*hin*-5717::FRT *fliD*23314::HiBiT (∆aa205-209, 3×SAGASA-HiBiT-3×SAGASA) ∆*flgKL*5739::FRT *fliI*23025 (E215Q) | This study |
| EM10744 | ∆*hin*-5717::FRT *fliC*23299::HiBiT (∆aa201-213 3×SAGASA-HiBiT-3×SAGASA) ∆*flgKL*5739::FKF | (3) |
| EM10852 | ∆*hin*-5717::FRT *fliC*23299::HiBiT (∆aa201-213 3×SAGASA-HiBiT-3×SAGASA) *fliI*23023 (R177H) ∆*flgKL*5739::FKF | This study |
| EM10853 | ∆*hin*-5717::FRT *fliC*23299::HiBiT (∆aa201-213 3×SAGASA-HiBiT-3×SAGASA) *fliI*23024 (G183A) ∆*flgKL*5739::FKF | This study |
| EM10854 | ∆*hin*-5717::FRT *fliC*23299::HiBiT (∆aa201-213 3×SAGASA-HiBiT-3×SAGASA) *fliI*23209 (E211D) ∆*flgKL*5739::FKF | This study |
| EM10950 | ∆*hin*-5717::FRT *fliC*23299::HiBiT (∆aa201-213 3×SAGASA-HiBiT-3×SAGASA) ∆*fliI*7364 ∆*flgKL*5739::FKF | This study |
| EM10951 | ∆*hin*-5717::FRT *fliC*23299::HiBiT (∆aa201-213 3×SAGASA-HiBiT-3×SAGASA) *fliI*23025 (E215Q) ∆*flgKL*5739::FKF | This study |
| EM10952 | ∆*hin*-5717::FRT *fliC*23299::HiBiT (∆aa201-213 3×SAGASA-HiBiT-3×SAGASA) *fliI*23210 (E211Q) ∆*flgKL*5739::FKF | This study |
| TH5633 | P*_flhDC_*5451::Tn10dTc[del-25] / pRG19 (P*_motA_*-*luxCDABE*, Cm^R^, Tet^R^) | (4) |
| TH14117 | P*_flhDC_*5451::Tn10dTc[del-25] *flgE*2219(T149N) / pRG19 (P*_motA_*-*luxCDABE*, Cm^R^, Tet^R^) | (4) |
| EM9611 | ∆*fliI*7364 P*_flhDC_*5451::Tn10dTc[del-25] / pRG19 (P*_motA_*-*luxCDABE*, Cm^R^, Tet^R^) | This study |
| EM9612 | *fliI*23023 (R177H) P*_flhDC_*5451::Tn10dTc[del-25] / pRG19 (P*_motA_*-*luxCDABE*, Cm^R^, Tet^R^) | This study |
| EM9613 | *fliI*23024 (G183A) P*_flhDC_*5451::Tn10dTc[del-25] / pRG19 (P*_motA_*-*luxCDABE*, Cm^R^, Tet^R^) | This study |
| EM9614 | *fliI*23025 (E215Q) P*_flhDC_*5451::Tn10dTc[del-25] / pRG19 (P*_motA_*-*luxCDABE*, Cm^R^, Tet^R^) | This study |
| EM11049 | *fliI*23209 (E211D) P*_flhDC_*5451::Tn10dTc[del-25] / pRG19 (P*_motA_*-*luxCDABE*, Cm^R^, Tet^R^) | This study |
| EM11050 | *fliI*23210 (E211Q) P*_flhDC_*5451::Tn10dTc[del-25] / pRG19 (P*_motA_*-*luxCDABE*, Cm^R^, Tet^R^) | This study |
| EM9031 | ∆*hin*-5717::FRT *fliC*6500(T237C) *flgE*7742::3×HA (after aa241) | Lab collection |
| EM12038 | *fliI*23023 (R177H) ∆*hin*-5717::FRT *fliC*6500(T237C) *flgE*7742::3×HA (after aa241) | This study |
| EM9688 | *fliI*23024 (G183A) ∆*hin*-5717::FRT *fliC*6500(T237C) *flgE*7742::3×HA (after aa241) | This study |
| EM11123 | ∆*hin*-5717::FRT *fliC*6500(T237C) *flgE*7742::3×HA (after aa241) *fliI*23025 (E215Q) | This study |
| EM11124 | ∆*hin*-5717::FRT *fliC*6500(T237C) *flgE*7742::3×HA (after aa241) *fliI*23209 (E211D) | This study |
| EM11125 | ∆*hin*-5717::FRT *fliC*6500(T237C) *flgE*7742::3×HA (after aa241) *fliI*23210 (E211Q) | This study |
| TH3730 | P*_flhDC_*5451::Tn10dTc[del-25] | (5) |
| EM9565 | *fliI*23023 (R177H) P*_flhDC_*5451::Tn10dTc[del-25] | This study |
| EM9566 | *fliI*23024 (G183A) P*_flhDC_*5451::Tn10dTc[del-25] | This study |
| **Plasmid** | **Genotype** | **Source** |
| pRG19 | P*_motA_*-*luxCDABE*, Cm^R^, Tet^R^ | (6) |
| pSUP | P*_glnS‘_*-BpaRS, P*_proK_*-6TRN, Cm^R^ | (7) |
| pEM9411 | pSUMO-*fliI* (full length), Kan^R^ | This study |
| EM11139 | pSUMO-*fliI* (full length, R177H), Kan^R^ | This study |
| EM11140 | pSUMO-*fliI* (full length, G183A), Kan^R^ | This study |
| EM11141 | pSUMO-*fliI* (full length, E211D), Kan^R^ | This study |
| EM11142 | pSUMO-*fliI* (full length, E211Q), Kan^R^ | This study |
| EM11146 | pSUMO-*fliI* (full length, E215Q), Kan^R^ | This study |
| pEM14338 | pTrc99A-FF4-*fliI* (D341amber)-SAGASA-3×FLAG, C-ter, Amp^R^ | This study |
| pEM15178 | pTrc99A-FF4-*fliI* (∆aa2-7, D341amber)-SAGASA-3×FLAG, C-ter, Amp^R^ | This study |
| pEM15179 | pTrc99A-FF4-*fliI* (R177H, D341amber)-SAGASA-3×FLAG, C-ter, Amp^R^ | This study |
| pEM15180 | pTrc99A-FF4-*fliI* (G183A, D341amber)-SAGASA-3×FLAG, C-ter, Amp^R^) / pSUP (artificial amino-acid, Cm^R^) | This study |
| pEM15181 | pTrc99A-FF4-*fliI* (E211D, D341amber)-SAGASA-3×FLAG, C-ter, Amp^R^) / pSUP (artificial amino-acid, Cm^R^) | This study |
| pEM15182 | pTrc99A-FF4-*fliI* (E211Q, D341amber)-SAGASA-3×FLAG, C-ter, Amp^R^ | This study |
| pEM15183 | pTrc99A-FF4-*fliI* (E215Q, D341amber)-SAGASA-3×FLAG, C-ter, Amp^R^ | This study |

**Table S3.** Oligonucleotides used in this study.

| **Sequence** | **Source** | **Identifier** |
| --- | --- | --- |
| GGATTCTGTGGCGCAAAG | Lab Collection | 5'-fliFseq4-fw |
| tggttttcaacgatggcgttagc | Lab Collection | 3'-fliK95-YscP(138-353)_gBlock_rv |
| ATTACCGGCACGCTCGAC | Lab Collection | 3'-FliI-int900_rv |
| CCTGAAACCAAAGAGGTGGA | Lab Collection | 5'-FliI-int180_fw |
| TTACGCGAGAAGTTCCTGCG | Lab Collection | 5'_fliG-Cter-fw |
| CGCTCAATAATGCAGGTGG | Lab Collection | 3'-FliH-sequencing-rev |
| aaccggcgcgttaatcacgccgccgtttaacccgctacagAGGGTTTTCCCAGTCACGAC | Lab Collection | 5'_FliI-dR152-F181-KanSceI_fw |
| tcatgccaagcagaaccgatttaccaacgccggaaccggcTGCTTCCGGCTCGTATGTTG | Lab Collection | 3'_FliI-dR152-F181-KanSceI_rv |
| ctacacgcgggcggacgtgattgtcgtgggacttatcggcAGGGTTTTCCCAGTCACGAC | Lab Collection | 5'_fliI_KanSceI_dE211_fw |
| gaccgtcggggccgagaatattttcgataaaatctttaacTGCTTCCGGCTCGTATGTTG | Lab Collection | 3'_fliI_KanSceI_dE215_rv |
| TATTATTACGCCATCCGAGCGTATGCGTCGTTTGAGTCGTagggttttcccagtcacgac | Lab Collection | FliN-KanSceI-Cter_FW |
| GCGGTGGGCTGAGAAACCGTGGCTTCTGTCTTCATCATTAtgcttccggctcgtatgttg | Lab Collection | FliN-KanSceI-Cter_RV |
| gtacttcgcgggtggagag | This study | fliG-int-rev2 |
| aagagttgtgtcgcctggcggcgccgggagtgctctgATGagggttttcccagtcacgac | This study | FliI-N-ter-KanSce-fw |
| TTTTGGCTTCAAAGTTGTCGAGCGCGGTAAGCCAGCGGGTtgcttccggctcgtatgttg | This study | FliI-N-ter-KanSce-rev |
| CCATTGCCTAGTCGGCACGCGCAATCCTCGACGGG | This study | fliI D341amber fw |
| GCGTGCCGACTAGGCAATGGGATCTTGTTGGTCGTCCCCCTC | This study | fliI D341amber rev |
| ggaattgtgagcggataacaatttcacacaggaaacagcaATGACCACGCGCCTGACC | This study | GA_fliI_fw |
| agccaagcttgcatgcctgcaggtcgactctagagTCACTTGTCATCATCATCTTTATAA | This study | GA_fliI-3XFLAG_rev |
| ATGATGATGACAAGTGAgatcctctagagtcgacctgcag | This study | GA_pTrc-fliI_fw |
| GGTCAGGCGCGTGGTCATtgctgtttcctgtgtgaaattgttatcc | This study | GA_pTrc-fliI-3XFLAG_rev |
| gtgattggcagcggcgaggatacctatgtctaatgaattgagggttttcccagtcacgac | This study | DfliH-KanSceI-fw |
| CAGCGGGTCAGGCGCGTGGTCATcagagcactcccggcgctgcttccggctcgtatgttg | This study | DfliH-KanSceI-rev |
| GTGAtaaagcaggagggcgacgatcatggcacaacatggcagggttttcccagtcacgac | This study | DfliJ-KanSceI-fw |
| gtgatcagttggggcagggtgatcattcgggtttcctcattgcttccggctcgtatgttg | This study | DfliJ-KanSceI-rev |
| CCGACGGTGTGATAAAGCAGG | This study | DfliJ fw |
| CGGTCAGcatATGGGACTCTTTGCCGGTTCCGGCGTTGGTAAATCGG | This study | fliI-R177H onestep fw |
| GAGTCCCATatgCTGACCGCGCCCTACGGTTAACAACGCGTTGATAGCGC | This study | fliI-R177H onestep rev |
| TTTGCCgcgTCCGGCGTTGGTAAATCGGTTCTGCTTGGCATGATGGCGCGC | This study | fliI-G183A onestep fw |
| CAACGCCGGAcgcGGCAAAGAGTCCCATacgCTGACCGCGCCC | This study | fliI-G183A onestep rev |
| GGGACTTATCGGCgatCGTGGCCGCgaaGTTAAAGATTTTATCGAAAATATTCTCGGCCC | This study | fliI-E211D onestep fw |
| CACGATCGCCGATAAGTCCCACGACAATCACGTCCGCCCGC | This study | fliI-E211D onestep rev |
| GGGACTTATCGGCcagCGTGGCCGCgaaGTTAAAGATTTTATCGAAAATATTCTCGGCCC | This study | fliI-E211Q onestep fw |
| CACGctgGCCGATAAGTCCCACGACAATCACGTCCGCCCGC | This study | fliI-E211Q onestep rev |
| CGTGGCCGCcagGTTAAAGATTTTATCGAAAATATTCTCGGCCCCGACGGTCG | This study | fliI-E215Q onestep fw |
| CTTTAACctgGCGGCCACGTTCGCCGATAAGTCCCACGACAATCACGTCC | This study | fliI-E215Q onestep rev |
| gaaacagcaATGTGGCTTACCGCGCTCGACAACTTTGAAGCCAAAATGGCGTTATTG | This study | fliI-Daa2-7 one-step fw |
| CGGTAAGCCACATtgctgtttcctgtgtgaaattgttatccgctcacaattccacacatt | This study | fliI-Daa2-7 one-step rev |
| aagagttgtgtcgcctggcggcgccgggagtgctctgATGTGGCTTACCGCGCTCGAC | This study | fliI-Daa2-7 replacement fw |

**Supplementary References**

1. Lee, HJ, Hughes, KT. 2006. Posttranscriptional control of the *Salmonella enterica* flagellar hook protein FlgE. J. Bacteriol. 188:3308–3316.

2. Erhardt, M, Mertens, ME, Fabiani, FD, Hughes, KT. 2014. ATPase-Independent Type-III Protein Secretion in *Salmonella enterica*. PLoS Genet. 10:e1004800.

3. Einenkel, R, Halte, M, Erhardt, M. *In* Journet, L, Cascales, E (ed), Quantifying Substrate Protein Secretion via the Type III Secretion System of the Bacterial Flagellum Bacterial Secretion Systems, p. 577–592. Bacterial Secretion Systems: Methods and Protocols, Humana, New York, NY.

4. Erhardt, M, Hirano, T, Su, Y, Paul, K, Wee, DH, Mizuno, S, Aizawa, S, Hughes, KT. 2010. The role of the FliK molecular ruler in hook-length control in *Salmonella enterica*. Mol. Microbiol. 75:1272–1284.

5. Karlinsey, JE, Tanaka, S, Bettenworth, V, Yamaguchi, S, Boos, W, Aizawa, S-I, Hughes, KT. 2000. Completion of the hook–basal body complex of the *Salmonella typhimurium* flagellum is coupled to FlgM secretion and *fliC* transcription. Mol. Microbiol. 37:1220–1231.

6. Goodier, RI, Ahmer, BM. 2001. SirA orthologs affect both motility and virulence. J. Bacteriol. 183:2249–2258.

7. Ryu, Y, Schultz, PG. 2006. Efficient incorporation of unnatural amino acids into proteins in *Escherichia coli*. Nat. Methods 3:263–265.
